# HD‐tDCS Restores Perivascular AQP4 Polarization via PPARγ Modulation to Enhance Glymphatic Clearance After Intracerebral Hemorrhage in Mice

**DOI:** 10.1002/advs.76660

**Published:** 2026-07-17

**Authors:** Zhiming Li, Yingmei Zhang, Qianqian Tong, Zhitao Gong, Shanyu Zhang, Zhen Qian, Hui Zhang, Qian Yao, Qi Li

**Affiliations:** ^1^ Department of Neurology The Second Affiliated Hospital of Anhui Medical University Hefei China; ^2^ Department of Rehabilitation Medicine The Second Affiliated Hospital of Anhui Medical University Hefei Anhui China; ^3^ School of Public Health Anhui Medical University Hefei Anhui China; ^4^ School of Mental Health and Psychological Sciences Anhui Medical University Hefei Anhui China

**Keywords:** astrocytes, brain edema, glymphatic system, intracerebral hemorrhage, transcranial direct current stimulation

## Abstract

Impaired perivascular aquaporin 4 (AQP4) polarization and glymphatic dysfunction after intracerebral hemorrhage (ICH) may delay hematoma and perihematomal edema resolution. The effects of high‐definition transcranial direct current stimulation (HD‐tDCS) on glymphatic transport and recovery after ICH, as well as the underlying mechanisms, are investigated in a collagenase‐induced mouse model. HD‐tDCS (anodal stimulation, 0.1 mA, 10 min daily) significantly enhances cerebrospinal fluid influx, improves interstitial solute clearance, reduces intracerebral tracer retention, and increases drainage to the deep cervical lymph nodes, as assessed by in vivo two‐photon imaging, contrast‐enhanced MRI, and ex vivo tracer analysis. HD‐tDCS also accelerates hematoma and edema resolution, reduces midline shift and diffusion abnormalities, and improves neurological outcomes. Mechanistically, ICH induces astrocytic proinflammatory activation together with impaired perivascular AQP4 polarization, whereas HD‐tDCS upregulates peroxisome proliferator‐activated receptor gamma (PPARγ), suppresses proinflammatory astrocyte activation, and restores perivascular AQP4 localization. Astrocyte‐specific knockdown or pharmacologic inhibition of PPARγ attenuates HD‐tDCS‐induced AQP4 repolarization, glymphatic recovery, and neurological improvement. These findings indicate that HD‐tDCS promotes hematoma and edema resolution after ICH in association with PPARγ‐dependent astrocyte remodeling, AQP4 repolarization, and glymphatic restoration.

## Graphical abstract

HD‐tDCS promotes recovery after intracerebral hemorrhage by enhancing PPARγ‐dependent astrocyte remodeling in a mouse model of ICH. This process suppresses proinflammatory astrocytic activation, restores perivascular AQP4 repolarization, and improves glymphatic influx and efflux, thereby facilitating hematoma and perihematomal edema clearance and ultimately improving neurological and behavioral outcomes after hemorrhagic brain injury.

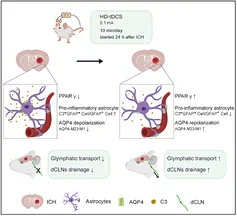

## 1 Introduction

Intracerebral hemorrhage (ICH), the most lethal and disabling stroke subtype, has adverse outcomes primarily determined by hematoma formation [1]. Hematoma mass effect and breakdown product driven inflammatory and coagulation cascades precipitate blood brain barrier (BBB) disruption, cerebral edema, and neuronal death [2]. Enhancing endogenous brain clearance pathways is a promising strategy to improve ICH outcomes [3].

Endogenous brain clearance depends on both glymphatic transport and downstream lymphatic drainage pathways [4]. The glymphatic system, formed by perivascular astrocytic endfeet, facilitates cerebrospinal fluid (CSF)—interstitial fluid (ISF) exchange and thereby supports metabolic waste clearance [5]. These solutes ultimately drain via meningeal lymphatic vessels or along the arachnoid sheath of the olfactory nerve to the cervical lymph nodes [5]. Central to this process is aquaporin‐4 (AQP4), densely localized on perivascular astrocytic endfeet [6]. Perivascular polarization of AQP4 is closely linked to efficient CSF–ISF exchange and solute clearance along perivascular pathways [7]. Restricting AQP4 expression or depolarizing its perivascular localization markedly impairs brain clearance.

Prior studies indicate that, after ICH, AQP4 depolarization coexists with glymphatic dysfunction and is associated with poor outcomes [8, 9]. Astrocyte activation has been linked to loss of AQP4 polarization [10]. Once AQP4 becomes depolarized, the resulting deficits in solute clearance can drive neuroinflammation, further compromising structures and functions associated with the glymphatic pathway [11]. Given the pivotal roles of astrocytes and AQP4 in glymphatic clearance, interventions targeting astrocyte‐associated AQP4 polarization to modulate glymphatic function hold therapeutic potential for ICH.

Transcranial direct current stimulation (tDCS), a noninvasive neuromodulation technique, has shown substantial promise in stroke treatment and rehabilitation, supported by multiple clinical studies and animal experiments [12, 13]. Prior work in healthy mice indicates that tDCS promotes CSF–ISF exchange but does not increase the perivascular polarization of AQP4 [14]. By contrast, mice with ICH exhibit AQP4 depolarization and structural disruption of perivascular spaces (PVS) [9]. It therefore remains important to determine whether HD‐tDCS can restore glymphatic function in the setting of ICH, where perivascular AQP4 localization is disrupted, and whether such effects are mediated by astrocyte remodeling.

In this study, we evaluated the effects of HD‐tDCS on glymphatic function and recovery from brain injury after ICH and examined the involvement of peroxisome proliferator activated receptor gamma (PPARγ)‐associated astrocyte remodeling, AQP4 repolarization, and glymphatic restoration in these effects. These findings reveal that the glymphatic system is a modifiable pathway for hematoma and edema clearance after intracerebral hemorrhage and suggest that HD‐tDCS may represent a potential therapeutic strategy for promoting recovery after ICH.

## 2 Materials and Methods

### 2.1 Animals

All experiments were performed on male C57BL/6 mice (8–10 weeks old; 23–25 g) purchased from GemPharmatech Co., Ltd. Mice were acclimated for at least 7 days prior to experimentation and housed in a specific pathogen‐free facility under a 12‐h light/dark cycle at 23 ± 1.5°C and 50 ± 10% relative humidity, with ad libitum access to standard chow and water; bedding and nesting materials were provided. Mice were randomly assigned to experimental groups according to the study design.

### 2.2 ICH Model and Drugs Administration

The mouse intracerebral hemorrhage model was established by collagenase injection as previously described [15]. For the validation experiments assessing MMP‐9/β‐dystroglycan (β‐DG)‐related remodeling, an autologous blood injection model of ICH was used [16]. GW9662 (HY‐16578, MCE), a PPARγ antagonist, was dissolved in DMSO and then diluted with phosphate‐buffered saline to a final concentration of 1% DMSO. To inhibit PPARγ activity, mice received daily intraperitoneal injections of GW9662 (1 mg/kg) after model induction until sacrifice. GW1929 (HY‐10648, MCE), a selective PPARγ agonist, was prepared similarly and administered intraperitoneally at 1 mg/kg after ICH induction until sacrifice.

### 2.3 MRI Experiments in Mice and Analysis

MRI data were acquired using a Bruker BioSpec 11.7‐T scanner (Bruker, Ettlingen, Germany). For T2‐weighted imaging, the acquisition parameters were as follows: matrix size, 256 × 256; field of view, 20 mm × 20 mm; repetition time (TR), 2500 ms; echo time (TE), 26 ms; slice thickness, 0.5 mm; 26 slices; and interslice gap, 0 mm. For diffusion weighted images (DWI), the sequence parameters are set as follows: matrix, 256 × 256; field of view, 20 mm × 20 mm; repetition time, 2500 ms; echo time, 16.5 ms; slice thickness, 0.5 mm; number of averages, 4. Apparent diffusion coefficient (ADC) maps were calculated using two b values (0 and 1000 s/mm^2^).

Contrast‐enhanced MRI was performed to assess brain‐wide glymphatic system function. Gadolinium‐diethylenetriamine pentaacetic acid (Gd‐DTPA, 6 µL) was injected into the cisterna magna at a rate of 1.2 µL/min. High‐resolution T1‐weighted images were acquired before contrast administration (baseline) and at 0.5, 2, 4, and 6 h after Gd‐DTPA injection. Imaging was performed using a rapid acquisition with relaxation enhancement sequence with the following parameters: matrix size, 256 × 256; field of view, 20 mm × 20 mm; TR, 400 ms; TE, 5.2 ms; and slice thickness, 0.8 mm. Both sagittal and axial images were acquired for contrast‐enhanced MRI analysis.

MRI images were analyzed using Image J. Based on anatomical landmarks, regions of interest (ROIs) were delineated on coronal sections, including the cortex, dorsal striatum, hippocampus, thalamus, and cerebellum. Mean signal intensity within each ROI was measured at each time point. Signal intensity was normalized to the preinjection baseline, and the percent signal change from baseline was calculated. Time–signal curves were then generated to characterize the distribution and clearance dynamics of Gd‐DTPA in different brain regions.

The area under the time–signal curve (AUC) was further calculated for each ROI to assess overall tracer distribution and glymphatic transport efficiency. To characterize influx and clearance kinetics, the initial rise slope (%/h) and the retention index were also derived from the time–signal curves. The initial rise slope was defined as the rate of signal increase during the early enhancement phase after contrast injection. The retention index was defined as the ratio of signal intensity at 6 h to the peak signal intensity (6 h/peak) and was used to reflect tracer retention and clearance efficiency within the brain. All ROI delineation and data extraction were performed by investigators blinded to group assignment.

### 2.4 Two‐Photon Imaging of CSF Tracer and Analysis

In vivo imaging of CSF tracer penetration into the brain parenchyma was performed using a Zeiss two‐photon microscope (LSM980 NLO) equipped with Airyscan. Mice with previously established thinned‐skull windows and cisterna magna cannulas were anesthetized with isoflurane, and their heads were secured under the microscope.

Immediately before imaging, 1 µL of 1% rhodamine B isothiocyanate‐dextran (RITC‐dextran; Sigma–Aldrich), dissolved in artificial cerebrospinal fluid (CSF), was injected into the cortex. To visualize the vasculature, 0.2 mL of 1% fluorescein isothiocyanate dextran (FITC‐dextran; Sigma–Aldrich), dissolved in saline, was administered intravenously immediately. A Zeiss 2‐photon imaging system (v3.9; ZEISS ZEN) equipped with a water immersion objective (20 ×, 1.0 N.A.) was used for imaging.

Image data were analyzed using NIH ImageJ software. To quantitatively assess the clearance of intracerebral tracer through the perivascular pathway after ICH, we selected stably traceable penetrating vessels in the ipsilateral cortex and performed the analysis on optical sections located approximately 100 µm below the cortical surface. Based on vascular outlines defined by the intravenously injected vascular tracer, regions of interest (ROIs) were placed around each target vessel, and the mean fluorescence intensity of intracortically injected RITC‐dextran was measured at 15, 30, 60, and 120 min. For each animal, three representative perivascular ROIs were selected, and their mean value served as the final measurement. Signals at each time point were normalized to the 15‐min value to characterize tracer clearance kinetics over time. Residual fluorescence intensities at 30, 60, and 120 min were further analyzed to assess between‐group differences in tracer clearance efficiency.

### 2.5 Ex Vivo Imaging of CSF Tracer and Analysis

For ex vivo assessment of CSF tracer influx, 0.5% Alexa Fluor 594 hydrazide (A594; Thermo Fisher Scientific) in 5 µL of artificial CSF was injected into the cisterna magna over 5 min, and brain tissue was collected 30 min later to evaluate tracer penetration into the parenchyma. Mean fluorescence intensity of A594 in brain sections was quantified as previously described. For each mouse, five 100‐µm‐thick coronal sections spanning −1.0 to +1.0 mm from bregma were analyzed, and tracer influx was measured and averaged to yield a single biological replicate.

To assess tracer drainage from the brain, a 1‐µL solution of 0.5% A594 and 0.5% FITC‐d2000 (2000 kDa; Thermo Fisher Scientific) in artificial CSF was injected into the striatum over 5 min, and brain tissue together with deep cervical lymph nodes (dCLNs) was collected 2 h later to evaluate tracer clearance from the parenchyma and drainage to the dCLNs. Quantification of A594 and FITC‐d2000 signals in brain sections and dCLN sections was performed as previously described. For each mouse, five 100‐µm‐thick coronal sections containing the injection site were analyzed for brain tracer retention, and the measured fluorescence values were averaged to generate a single biological replicate. For dCLN analysis, three 30‐µm‐thick sections from each animal were examined, and tracer fluorescence was measured and averaged for statistical analysis.

### 2.6 High‐Definition Transcranial Direct Current Stimulation Intervention

High‐definition transcranial direct current stimulation (HD‐tDCS) was used in this study. Stimulation was initiated 24 h after ICH induction and was then delivered once daily at the same time each day until euthanasia, for up to 7 sessions. For stimulation, mice were anesthetized with isoflurane using a gas anesthesia system and secured in a stereotaxic frame with the skull kept level. Mice assigned to the control groups underwent the same anesthesia and sham stimulation procedures on the same schedule. Hair over the scalp and back was shaved to expose the skin. A conductive medium composed of 1.5% agarose dissolved in phosphate‐buffered saline was applied over a circular area centered 2.00 mm posterior and 2.00 mm lateral to bregma, and the anodal electrode was positioned over this site. The cathodal electrode was placed over the exposed skin on the back. The stimulation parameters (0.1 mA, 10 min daily) were selected on the basis of previous rodent tDCS studies and preliminary optimization, which suggested that this protocol was sufficient to induce neuromodulatory effects without causing detectable tissue injury [14].

### 2.7 Brain Water Content Measurement

Brain water content was measured using the wet–dry weight method. At the indicated time points, mice were deeply anesthetized, and the brains were rapidly removed. The cortex, brainstem, cerebellum, basal ganglia, and hippocampus were carefully dissected on ice. Each tissue sample was immediately weighed to obtain the wet weight and then dried at 80°C until a constant dry weight was reached. Brain water content was calculated using the following formula: brain water content (%) = (wet weight − dry weight) / wet weight × 100%. Each dissected brain region from each animal was treated as one biological replicate for statistical analysis.

### 2.8 Laser Speckle Contrast Imaging of Cerebral Blood Flow (CBF)

CBF was assessed using laser speckle contrast imaging. Mice were anesthetized with isoflurane and placed on a temperature‐controlled heating pad to maintain body temperature at 37°C. After the scalp was gently exposed, the skull surface was kept intact and moist during imaging. Laser speckle images were acquired at the indicated time points. For quantitative analysis, a fixed ipsilateral ROI corresponding to the perihematomal area was selected. The same ROI size and anatomical location were applied across animals. Mean CBF values within the ipsilateral ROI were calculated and expressed as arbitrary perfusion units. For each animal, stable consecutive images were averaged to obtain one biological replicate. Image acquisition and ROI analysis were performed by investigators blinded to group assignment.

### 2.9 Behavioral Assessments

All behavioral tests were performed by investigators blinded to group assignment. To minimize the effects of environmental variation, all assessments were conducted at fixed time points during the day. Before formal testing, mice were allowed to acclimate to the testing environment for 30 min. Unless otherwise specified, behavioral evaluations were performed before surgery to obtain baseline measurements and repeated at the indicated time points after ICH.

### 2.10 Modified Neurological Severity Score (mNSS)

Neurological deficits were assessed using the modified neurological severity score (mNSS). This composite score evaluates multiple domains, including motor, sensory, reflex, and balance function, with higher scores indicating more severe neurological impairment. The test battery included assessment of forelimb and hindlimb motor abnormalities, sensory deficits, startle reflex, and beam‐walking performance. Each mouse was scored independently by 2 observers blinded to group allocation, and the mean score was used for subsequent analysis.

### 2.11 Rotarod Test

The rotarod test was used to evaluate motor coordination, balance, and endurance. Before formal testing, all mice underwent a 1‐week habituation period consisting of daily 30‐min training sessions, during which the rotation speed was gradually increased from 5 to 20 rpm to reduce anxiety and novelty‐related effects. On the test day, the maximum trial duration was set at 5 min, and the maximum rotation speed was limited to 40 rpm. During testing, each mouse was placed on the rotating rod, and the trial was terminated when the mouse either fell or clung to the rod for 2 consecutive rotations without active walking. The latency to fall and the corresponding speed were recorded. Each mouse was tested three times with an interval of 10–15 min between trials, and the mean value was used for statistical analysis.

### 2.12 Open Field Test

The open field test was used to assess spontaneous locomotor activity and anxiety‐like behavior. The apparatus consisted of an open‐field arena measuring 40 cm × 40 cm × 40 cm. During testing, each mouse was placed individually in the center of the arena and allowed to explore freely for 5 min. A video‐tracking system was used to record locomotor trajectories and automatically analyze total distance traveled, mean speed, number of entries into the center zone, and time spent in the center zone. After each trial, the arena was cleaned with 75% ethanol to eliminate residual odor cues.

### 2.13 Novel Object Recognition (NOR) Test

The novel object recognition test was used to assess recognition memory [17]. Before testing, mice were habituated to the empty open‐field arena to reduce anxiety and novelty‐related exploration. During the training phase, two identical objects were placed symmetrically in the arena, and each mouse was allowed to freely explore the objects. After 24 h, one familiar object was replaced with a novel object of similar size but different shape and appearance. The position of the novel object was counterbalanced across animals to avoid side preference. Exploration was defined as the mouse directing its nose toward the object within a short distance or physically sniffing the object. The arena and objects were cleaned with 75% ethanol between trials to remove olfactory cues. Recognition performance was quantified using the discrimination index(DI%): (time exploring the novel object − time exploring the familiar object) / (time exploring the novel object + time exploring the familiar object) × 100%.

### 2.14 Gait Analysis

Gait parameters were evaluated using the DigiGait Imaging System (Mouse Specifics Inc., USA). Before data acquisition, all mice underwent 1 week of treadmill habituation training. During this period, mice walked daily for 5 min at different speeds ranging from 8 to 15 cm/s to adapt to the testing environment and treadmill locomotion. Before formal testing, mice were acclimated in a room adjacent to the testing area for 30 min, then weighed, and tested. During acquisition, the treadmill speed was initially set at 8 cm/s, gradually increased to 12 cm/s, and maintained for 30 s for data collection. Testing was stopped after each mouse completed 3–5 valid runs. Gait parameters, including swing time, stance time, stride length, and paw area, were automatically extracted using the accompanying software and used to assess locomotor recovery.

### 2.15 RNA Sequencing and Bioinformatics Analysis

RNA sequencing was performed with technical support from Genesky Biotechnologies, Inc. (Shanghai, China). To investigate the molecular mechanisms associated with HD‐tDCS intervention after ICH, 3 brain tissue samples were randomly selected from each of the SHAM, ICH, and ICH+tDCS groups for RNA sequencing. Perihematomal brain tissue was collected at the designated time point, and total RNA was extracted using TRIzol reagent (Invitrogen, CA, USA). RNA concentration and purity were assessed using a NanoDrop ND‐1000 spectrophotometer (NanoDrop, Wilmington, DE, USA), and samples that met library preparation requirements were used for subsequent sequencing.

After library construction, paired‐end sequencing (PE150 mode) was performed on the Illumina NovaSeq 6000 platform (Illumina, USA). Raw sequencing data were first evaluated using FastQC v0.11.4, including assessment of per‐base sequence quality, GC content, sequence duplication levels, and adapter contamination. Residual adapters, low‐quality bases, and short reads were then removed using fastp v0.19.5 to generate clean reads. Standard quality‐control metrics, including total read counts, Q30 percentages, mapping rates, and sample clustering, were further examined.

Clean reads were aligned to the mouse reference genome GRCm39/mm39 using STAR v2.7.7a, and transcript abundance was quantified with StringTie v1.3.5. Differential expression analysis was performed using DESeq2 v1.10.1, with false discovery rate (FDR) correction by the Benjamini–Hochberg method. Genes with |log2(fold change)| > 1 and adjusted *p* < 0.05 were considered differentially expressed.

To further characterize the biological functions of the differentially expressed genes, clusterProfiler v2.4.2 was used for gene set enrichment analysis (GSEA), Gene Ontology (GO) enrichment analysis, and Kyoto Encyclopedia of Genes and Genomes (KEGG) pathway enrichment analysis. Enrichment analysis was performed using Fisher's exact test with FDR correction.

### 2.16 Immunogold Electron Microscopy

After transcardial perfusion with PBS followed by 4% paraformaldehyde (PFA), brains were collected and postfixed overnight in 4% PFA at 4°C. Brain tissue was then treated with 0.02 mol/L glycine and cut into 40‐µm sections using a vibratome. Sections were permeabilized with 0.1% Triton X‐100, blocked with 1% bovine serum albumin (BSA), and incubated overnight at 4°C with a rabbit anti‐AQP4 primary antibody. Immunolabeling was then performed using a colloidal gold–conjugated secondary antibody, followed by silver enhancement when necessary. After labeling, sections were postfixed with 0.5% osmium tetroxide, dehydrated through graded ethanol, transitioned through propylene oxide, and embedded in TAAB Epon, followed by polymerization at 60°C for 48 h. Ultrathin sections (approximately 60 nm) were stained with lead citrate and examined using a transmission electron microscope. The distribution of AQP4 immunogold particles on perivascular astrocytic endfoot membranes and within the cytoplasm was analyzed.

### 2.17 Immunofluorescence Staining and Analysis

After perfusion with PBS and 4% paraformaldehyde, brains were collected, postfixed overnight at 4°C, cryoprotected, and cut into frozen sections. Sections were permeabilized with Triton X‐100, blocked with bovine serum albumin, and incubated overnight at 4°C with primary antibodies: rabbit anti‐AQP4(1:500; Abcam, ab259318), mouse anti‐GFAP (1:500; CST, 3670), rabbit anti‐C3 (1:500; Abcam, ab97462), rabbit anti‐S100A10 (1:500; Invitrogen, PA5‐95505), rabbit anti‐CD31 (1:500; Abcam, ab182981), rabbit anti‐CD206 (1:500; Abcam, ab64693), rabbit anti‐IBA1 (1:500; Abcam, ab178846), rabbit anti‐CD86 (1:500; Proteintech, 13395‐1‐AP) and rabbit anti‐PPARγ (1:500; CST, 81B8). The next day, sections were incubated with the appropriate fluorescent secondary antibodies and counterstained with DAPI. Images were acquired using a confocal microscope and analyzed with ImageJ. AQP4 polarization and perivascular coverage were assessed by their perivascular enrichment [18], and the proportions of C3^+^GFAP^+^ and S100A10^+^GFAP^+^ cells were used to reflect distinct astrocyte phenotypes.

### 2.18 Western Blot

Perihematomal brain tissue was homogenized in RIPA lysis buffer containing protease and phosphatase inhibitors. After centrifugation, the supernatant was collected, and protein concentration was measured using a BCA assay. Equal amounts of protein were separated by SDS‐PAGE and transferred to PVDF membranes. After blocking, membranes were incubated overnight at 4°C with primary antibodies: rabbit anti‐PPARγ (1:1000; CST, 81B8), mouse anti‐AQP4 (1:200; Santa Cruz Biotechnology, sc‐390488), mouse anti‐GFAP (1:1000; CST, 3670), rabbit anti‐C3 (1:1000; Abcam, ab97462), rabbit anti‐S100A10 (1:1000; Invitrogen, PA5‐95505), rabbit anti‐NF‐κB (1:1000; CST, 8242), rabbit anti‐p‐NF‐κB (1:1000; CST, 3033), rabbit anti‐mTOR (1:1000; Affinity Biosciences, AF6308), rabbit anti‐p‐mTOR (1:1000; Affinity Biosciences, AF6308), rabbit anti‐MMP9 (1:1000; Proteintech, 30592‐1‐AP), rabbit anti‐β‐DG (1:200; Santa Cruz Biotechnology, sc‐33702). The following day, membranes were incubated with HRP‐conjugated secondary antibodies, visualized using ECL, and imaged. Rabbit anti‐β‐actin (1:3000; Affinity Biosciences, AF7018) or rabbit anti‐GAPDH (1:3000; Affinity Biosciences, AF7021) was used as the loading control, and band intensity was quantified with ImageJ.

### 2.19 RT‐qPCR

Total RNA was extracted from perihematomal brain tissue using TRIzol, and cDNA was synthesized by reverse transcription. Real‐time quantitative PCR was performed to measure total AQP4, AQP4‐M1, AQP4‐M23, C3, Serping1, Ggta1, S100a10, Clcf1, and Ptgs2. GAPDH or β‐actin was used as the internal control, and relative expression levels were calculated using the 2^‐ΔΔCt method. Primer sequences are provided in Table S1.

### 2.20 Quantification and Statistical Analysis

Statistical analysis was performed using GraphPad Prism 10. Data were presented as mean ± SEM. Sample sizes for imaging, molecular, and behavioral experiments were initially determined with reference to previous studies [19] and were further evaluated by sensitivity analyses using the actual biological sample sizes, with a two‐sided α of 0.05 and a target power of 0.80 [20]; representative analyses are summarized in Table S2. RNA‐seq with *n* = 3/group was considered exploratory, and for imaging or fluorescence quantifications, multiple fields, sections, or ROIs from the same animal were treated as technical sampling and averaged to generate one animal‐level value before statistical testing. No successfully collected data or animals were excluded. Data normality was evaluated with the Shapiro–Wilk test. Comparisons between 2 groups were performed using an unpaired 2‐tailed Student's *t* test for normally distributed data or the Wilcoxon rank‐sum test for nonnormally distributed data. For multiple‐group comparisons, 1‐way or 2‐way ANOVA followed by Bonferroni's multiple‐comparison test was used as appropriate. Repeated measurements over time were analyzed using 2‐way repeated‐measures ANOVA. Correlations were assessed using Pearson or Spearman methods, as appropriate. A 2‐sided *p* value < 0.05 was considered statistically significant.

## 3 Results

### 3.1 HD‐tDCS Promotes Glymphatic Influx and Clearance in ICH Mice

First, we used in vivo two‐photon microscopy to determine whether HD‐tDCS improved glymphatic clearance in the ipsilateral cortex after ICH. As shown in Figure 1A, RITC‐dextran was injected into the cortex, followed by tail‐vein injection of FITC‐dextran to label the vasculature. Tracer clearance was assessed by quantifying the relative fluorescence intensity of RITC‐dextran in the PVS at each time point, normalized to the intensity measured at 15 min after injection. At 30 min after injection, no significant difference in RITC‐dextran clearance was observed among the three groups, although signal intensity gradually declined from 15 to 120 min in all groups. However, at both 30 and 120 min after RITC injection, RITC‐dextran clearance was significantly lower in the ICH group than in the sham group, and was also lower than that in the ICH + tDCS group at 120 min (Figure 1B,C). These findings indicate that glymphatic clearance is impaired after ICH, whereas HD‐tDCS facilitates the recovery of glymphatic clearance function.

**FIGURE 1:**
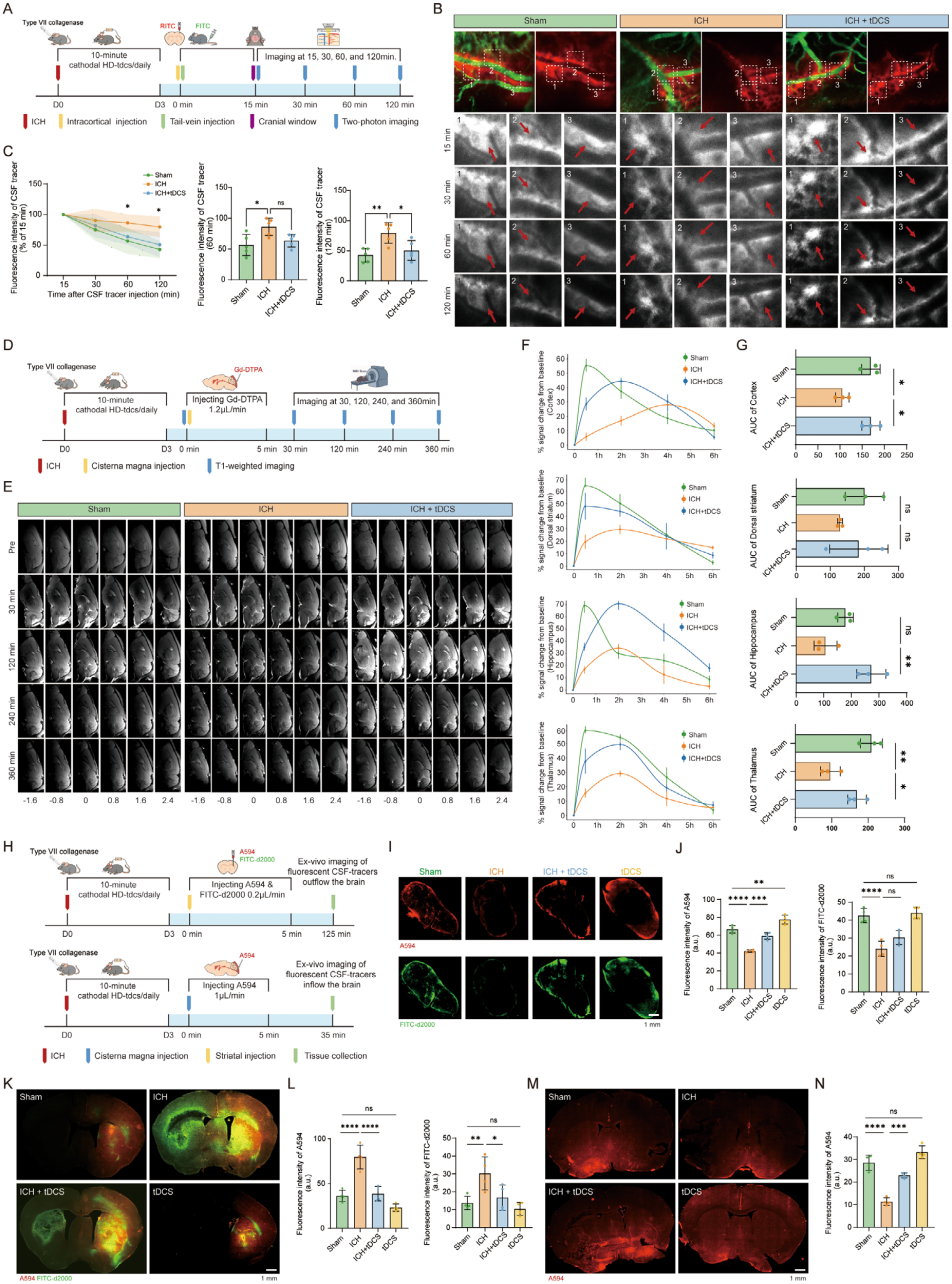
HD‐tDCS improves glymphatic transport in ICH mice. (A) Experimental timeline of two‐photon images. (B) Representative two‐photon time‐lapse images showing RITC‐dextran tracer distribution and clearance within PVS after tracer injection. Red arrows indicate representative tracer signals in PVS. (C) The fluorescence intensity of RITC‐dextran was quantitatively analyzed, showing a dramatic impairment in the tracer clearance in PVS (scale bar, 50 µm) (*n* = 5). (D) Experimental timeline of contrast‐enhanced MRI. (E) Representative MRI sagittal images of early influx and late efflux of the Gd‐DTPA along the paravascular pathway over 6 h (pre, 0.5, 2, 4, and 6 h). (F, G) Time signal curves and AUC of Gd‐DTPA enhancement in mouse cortex, dorsal striatum, hippocampus, and thalamus (*n* = 3). (H) Experimental timelines for ex vivo fluorescent tracer analysis of glymphatic outflow and influx. (I, J) The representative dCLNs fluorescence images display the efflux of mouse brain parenchyma A594 and FITC‐d2000, and quantitatively analyze the fluorescence intensity (scale bar, 1 mm) (*n* = 4). (K, L) Representative coronal brain slices showed that A594 and FITC‐d2000 were retained after injection of tracer into the striatum, and quantitative analysis of the fluorescence intensity was performed to evaluate CSF efflux (scale bar, 1 mm) (*n* = 4). (M, N) Representative coronal brain slices showed that A594 was retained after injection of tracer into the cisterna magna, and quantitative analysis of the fluorescence intensity was performed to evaluate CSF influx (scale bar, 1 mm) (*n* = 4). Data are presented as mean ± SEM. ns, not significant; ^*^ *p* < 0.05, ^**^ *p* < 0.01, ^***^ *p* < 0.001, ^****^ *p* < 0.0001.

To evaluate the effect of HD‐tDCS on glymphatic transport in ICH mice, Gd‐DTPA was injected into the cisterna magna, and contrast‐enhanced MRI was performed to monitor the distribution of the Gd‐DTPA in the brain at 30 min, 2 h, 4 h, and 6 h after injection (Figure 1D).

Consistent with previous observations, Gd‐DTPA administered into the cisterna magna migrated along the basilar artery, traversed the pituitary region, and reached the olfactory bulb via the olfactory artery, before gradually extending from the cortical surface into the deeper brain parenchyma [21]. Widespread brain enhancement was observed from 30 min to 2 h after injection, with the strongest signal detected in the cerebellum. Thereafter, signal intensity declined in most regions after 2 h (Figure 1E).

Analysis of signal intensity across different brain regions showed that, in the Sham group, Gd‐DTPA signals in the cortex, dorsal striatum, hippocampus, and thalamus exhibited a characteristic temporal pattern, with a rapid early increase followed by a gradual decline. In contrast, the ICH group showed an overall attenuation of signal enhancement in these regions, accompanied by lower peak values and a delayed time to peak, indicating impaired transport of cerebrospinal fluid into the brain parenchyma after ICH. Following HD‐tDCS treatment, the time–signal curves in the cortex, hippocampus, and thalamus were partially restored compared with those in the ICH group, as reflected by stronger early enhancement, an earlier peak, and greater overall tracer distribution, with the most pronounced improvement observed in the hippocampus (Figure 1F).

Further analysis of the area under the curve (AUC) showed that, compared with the Sham group, the ICH group had significantly lower AUC values in the cortex and thalamus. Relative to the ICH group, ICH + HD‐tDCS group significantly increased the AUC values in the cortex, hippocampus, and thalamus, with the most prominent increase observed in the hippocampus (Figure 1G). In contrast, the cerebellum showed a broadly similar time–signal pattern across the ICH + HD‐tDCS groups, with no evident between‐group differences (Figure S1A). Consistently, analysis of the initial rise slope and retention index further supported region‐specific alterations in tracer influx and clearance kinetics across groups (Figure S1B,C). These findings suggest that ICH disrupts glymphatic transport in multiple brain regions, as reflected by reduced tracer distribution and delayed transport kinetics, whereas HD‐tDCS treatment partially reverses these changes, with more pronounced effects in the cortex, hippocampus, and thalamus.

To further evaluate the effects of HD‐tDCS on glymphatic transport after ICH, we used ex vivo fluorescence tracer imaging to examine both glymphatic clearance and influx (Figure 1H). Following intrastriatal injection of A594 and FITC‐d2000, tracer fluorescence in the deep cervical lymph nodes (dCLNs) was markedly reduced in the ICH group compared with the Sham group, whereas tracer retention within the brain parenchyma was increased, indicating impaired efflux of interstitial solutes after ICH. HD‐tDCS treatment significantly increased tracer signals in the dCLNs and reduced parenchymal retention, suggesting partial restoration of glymphatic efflux (Figure 1I–L). To further determine whether this effect was already present at an earlier stage, we additionally performed the same ex vivo tracer efflux assay on Day 1 after ICH. Consistent with the findings observed at Day 3 after ICH, the ICH group showed reduced tracer drainage to the dCLNs and increased intracerebral tracer retention on Day 1, whereas HD‐tDCS treatment enhanced tracer drainage and reduced brain tracer retention (Figure S1D–G). After intracisternal injection of A594, fluorescence intensity within the brain parenchyma was clearly decreased in the ICH group, but was restored following HD‐tDCS treatment (Figure 1M,N), indicating that HD‐tDCS also promoted cerebrospinal fluid influx into the brain after ICH. Taken together, these findings indicate that HD‐tDCS alleviates both influx and efflux deficits of the glymphatic system after ICH.

### 3.2 HD‐tDCS Enhances Perivascular AQP4 Localization in ICH Mice

To investigate the molecular basis of HD‐tDCS‐mediated improvement in glymphatic function after ICH, we analyzed the differentially expressed genes identified in the Sham vs. ICH and ICH vs. ICH+tDCS comparisons. AQP4 was identified as a candidate molecule potentially involved in the effects of HD‐tDCS (Figure 2A). Although total AQP4 mRNA levels were elevated after ICH compared with the Sham group, HD‐tDCS did not significantly alter overall AQP4 transcription (Figure 2B–D).

**FIGURE 2:**
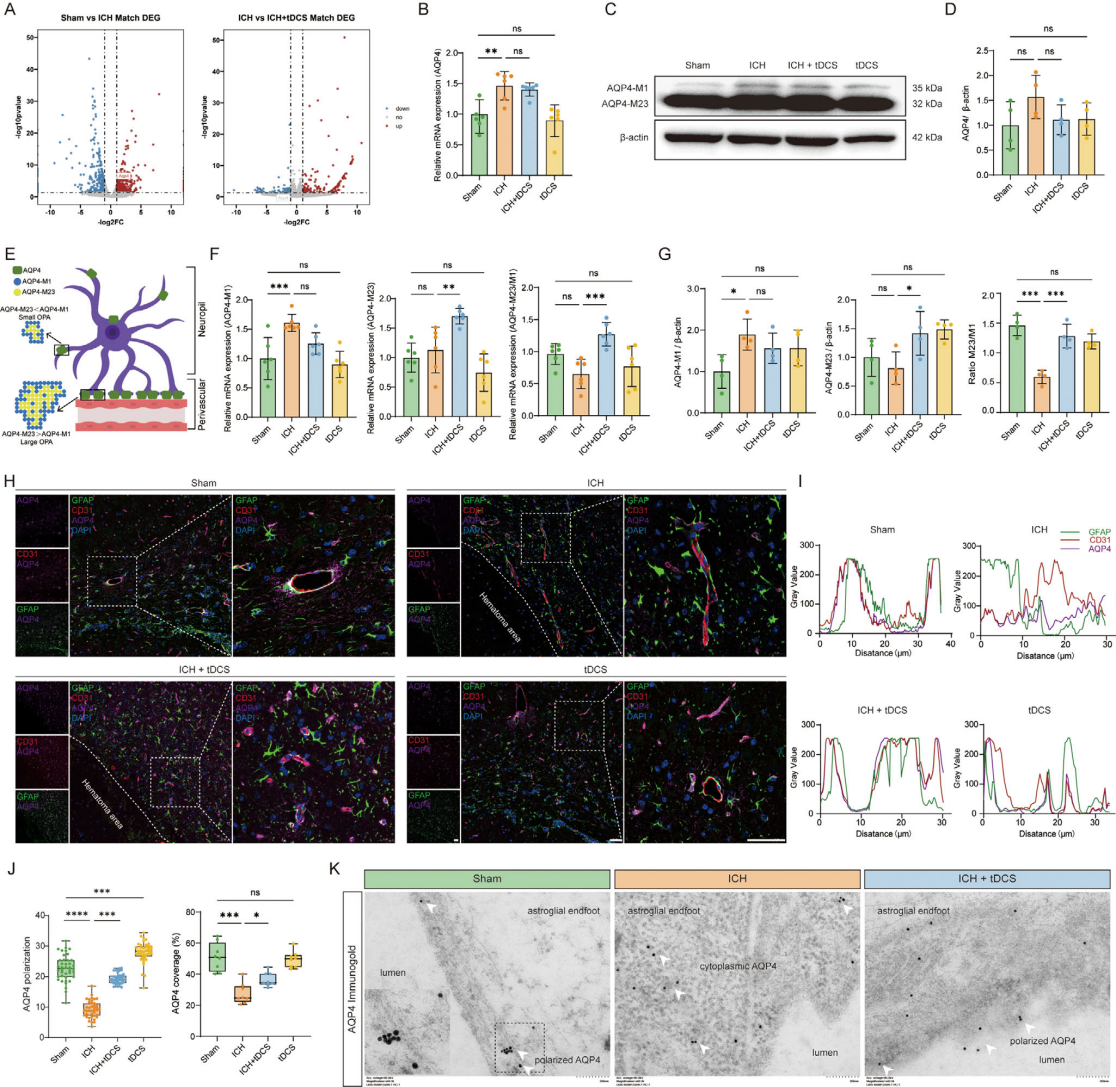
HD‐tDCS restores perivascular AQP4 polarization after ICH. (A) Volcano plots of differentially expressed genes in the comparisons of Sham vs. ICH and ICH vs. ICH+tDCS, identifying AQP4 as a potential candidate molecule. (B) Quantitative analysis of total AQP4 mRNA expression in each group (*n* = 4). (C) Representative Western blot bands of AQP4 protein expression in each group. (D) Quantitative analysis of AQP4 protein expression (*n* = 4). (E) Schematic diagram of AQP4‐M1 and AQP4‐M23 isoforms and their relationship to orthogonal arrays of particles (OAPs) and AQP4 polarization. (F) Quantitative analysis of AQP4‐M1 and AQP4‐M23 mRNA expression and the ratio of AQP4‐M23 to AQP4‐M1 (*n* = 4). (G) Quantitative analysis of AQP4‐M1 and AQP4‐M23 protein expression and the ratio of AQP4‐M23 to AQP4‐M1 (*n* = 4). (H) Representative triple immunofluorescence images of AQP4 (purple), GFAP (green), and CD31 (red) in the perihematomal region. Nuclei were counterstained with DAPI (blue). Merged images and higher‐magnification views show the distribution of AQP4 at perivascular astrocytic endfeet (scale bar, 50 µm). (I) Line‐scan analysis of GFAP, CD31, and AQP4 fluorescence intensity in perivascular regions in each group. (J) Quantitative analysis of AQP4 polarization and perivascular AQP4 coverage (*n* = 8). (K) Representative immunogold electron micrographs of AQP4 in perivascular astrocytic endfeet, showing redistribution of AQP4 from the endfoot membrane to the cytoplasm after ICH, which was partially reversed by HD‐tDCS. Data are presented as mean ± SEM. ns, not significant; ^*^ *p* < 0.05, ^**^ *p* < 0.01, ^***^ *p* < 0.001, ^****^ *p* < 0.0001.

Glymphatic function is highly dependent on the perivascular polarization of AQP4 [6, 7] (Figure 2E). Given the close association between AQP4 isoforms and its perivascular polarization, we next examined changes in AQP4‐M1 and AQP4‐M23 (Figure 2F,G). In perihematomal tissue, AQP4‐M1 mRNA levels increased after ICH, whereas the AQP4‐M23/M1 ratio decreased; following HD‐tDCS treatment, AQP4‐M23 expression increased, and the AQP4‐M23/M1 ratio was markedly restored (Figure 2F). Protein analysis showed that AQP4‐M1 was increased and the AQP4‐M23/M1 ratio was markedly decreased in the ICH group. HD‐tDCS increased AQP4‐M23 protein expression and the AQP4‐M23/M1 ratio, without altering total AQP4 protein levels (Figure 2G). These findings suggest that ICH disrupts the balance of AQP4 isoforms, shifting it toward a less polarized state, whereas HD‐tDCS treatment partially reverses this change.

Immunofluorescence further showed that, in the Sham group, AQP4 was predominantly enriched in GFAP‐positive astrocytic endfeet surrounding CD31‐positive blood vessels. In contrast, in perihematomal tissue from ICH mice, AQP4 staining was more diffusely distributed, with reduced perivascular enrichment. After HD‐tDCS treatment, perivascular AQP4 localization was partially restored, and line scan analysis further demonstrated improved spatial alignment with perivascular structures (Figure 2H,I). Quantitative analysis showed that AQP4 polarization and perivascular coverage were significantly reduced in ICH mice, whereas both measures were markedly increased after HD‐tDCS treatment (Figure 2J). Notably, the contralateral hemisphere showed a similar pattern, with reduced AQP4 polarization and decreased perivascular coverage in the ICH group, both of which were partially reversed by HD‐tDCS treatment (Figure S2). Immunogold electron microscopy further confirmed that, in the Sham group, AQP4 immunogold particles were primarily localized to the perivascular astrocytic endfoot membrane. In the ICH group, AQP4 labeling was reduced at perivascular endfeet but increased in astrocytic somata. After HD‐tDCS treatment, AQP4 distribution at perivascular endfeet was partially restored (Figure 2K). Therefore, our findings indicate that HD‐tDCS enhances perivascular AQP4 polarization by altering AQP4 isoform composition and promoting its relocalization to perivascular astrocytic endfeet.

### 3.3 HD‐tDCS Improves Neurological Recovery in ICH Mice

Based on the beneficial effects of HD‐tDCS on glymphatic transport and AQP4 polarization in ICH mice, we next examined its impact on hematoma and perihematomal edema evolution and neurological recovery after ICH (Figure 3A). Serial MRI showed time‐dependent changes in perihematomal edema, hematoma volume, and midline shift (MLS) after ICH. Edema peaked on day 3, whereas hematoma volume gradually declined after day 1 (Figure 3B–E). Compared with the ICH group, the ICH + tDCS group showed greater lesion improvement during recovery, with smaller edema and hematoma volumes on days 5 and 7 and less MLS on day 5 (Figure 3C–E). In addition, the relative apparent diffusion coefficient (rADC) was higher in the ICH + tDCS group than in the ICH group on day 3, suggesting that HD‐tDCS ameliorated diffusion abnormalities in perihematomal tissue and reduced cytotoxic edema after ICH (Figure 3F). In addition, we further measured brain water content in different brain regions on Days 1 and 3 after ICH. Although brain water content was increased after ICH, no significant difference in brain water content was observed between the ICH and ICH+tDCS groups at either time point (Figure S3A–D). This temporal pattern also raises the possibility that improved glymphatic clearance may contribute to subsequent hematoma and edema resolution, rather than being only a downstream result of reduced edema.

**FIGURE 3:**
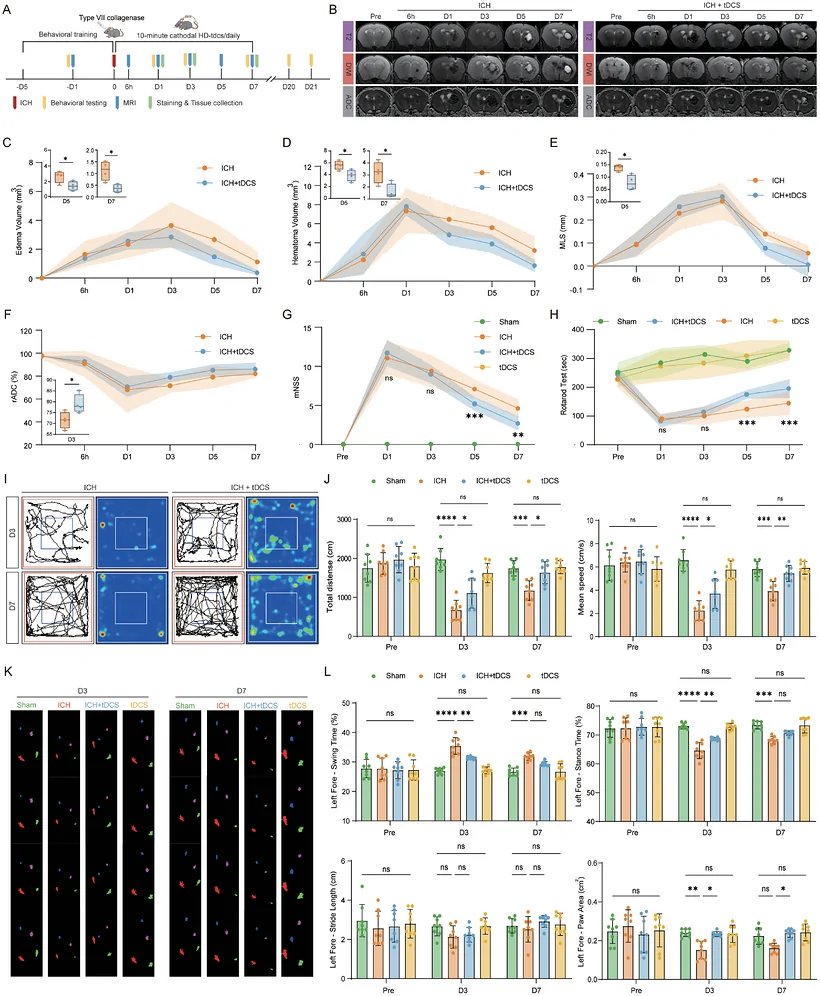
HD‐tDCS promotes hematoma and edema clearance and improves neurological function after ICH. (A) Schematic diagram of the experimental design. (B) Representative T2‐weighted, diffusion‐weighted imaging (DWI), and apparent diffusion coefficient (ADC) MRI images from the ICH and ICH+tDCS groups at Pre, 6 h, D1, D3, D5, and D7. (C) Quantitative analysis of perihematomal edema volume at different time points (*n* = 4). (D) Quantitative analysis of hematoma volume at different time points (*n* = 4). (E) Quantitative analysis of midline shift (MLS) at different time points (*n* = 4). (F) Quantitative analysis of relative apparent diffusion coefficient (rADC) at different time points (*n* = 4). (G) Modified neurological severity score (mNSS) in each group at different time points (*n* = 8). (H) Rotarod performance in each group at different time points (*n* = 8). (I) Representative locomotor trajectories and heat maps from the open‐field test in the ICH and ICH+tDCS groups at D3 and D7. (J) Quantitative analysis of total travel distance and mean speed in the open‐field test (*n* = 8). (K) Representative gait analysis images from each group at D3 and D7. (L) Quantitative analysis of left forelimb swing time, stance time, stride length, and paw area in each group (*n* = 8). Data are presented as mean ± SEM. ns, not significant; ^*^ *p* < 0.05, ^**^ *p* < 0.01, ^***^ *p* < 0.001, ^****^ *p* < 0.0001.

Behavioral assessments further supported a beneficial effect of HD‐tDCS on neurological recovery after ICH. mNSS scores showed that neurological deficits were most pronounced on day 1 after ICH and gradually improved thereafter, whereas HD‐tDCS treatment significantly reduced mNSS scores on days 5 and 7 (Figure 3G). In the rotarod test, ICH mice showed marked motor coordination deficits at early time points, whereas HD‐tDCS treatment significantly prolonged the latency to fall on days 5 and 7 (Figure 3H). In the open‐field test, mice in the ICH + tDCS group showed more active movement trajectories and hotspot distribution than those in the ICH group on days 3 and 7. Total travel distance and mean speed were also significantly increased, indicating improved spontaneous locomotor activity (Figure 3I,J).

Gait analysis showed a pattern consistent with the behavioral findings above (Figure 3K,L). Compared with the Sham group, ICH mice showed prolonged swing time, reduced stance time, and decreased paw area in the left forelimb. HD‐tDCS treatment shortened swing time and partially restored stance time and paw area (Figure 3L). Accordingly, HD‐tDCS treatment effectively mitigated brain injury and promoted neurological recovery in ICH mice.

To further assess whether the beneficial effects of HD‐tDCS persisted beyond the acute/subacute phase, we performed the novel object recognition test at 21 days after ICH (Figure S3E,F). During the test phase, ICH mice exhibited a significantly reduced discrimination index compared with Sham mice, whereas HD‐tDCS significantly increased the discrimination index after ICH, suggesting partial improvement of long‐term recognition memory. Although total exploration time was reduced after ICH and was not significantly restored by HD‐tDCS, the improvement in discrimination index suggests enhanced novel‐object discrimination rather than a nonspecific increase in exploratory activity.

### 3.4 HD‐tDCS Curbs Astrocytic Overactivation and Drives Astrocytes Toward AQP4 Polarization‐Linked Phenotypes

Because perivascular AQP4 polarization is closely tied to the structural and functional integrity of astrocytic endfeet, we further examined astrocyte activation, phenotypic changes, and their relationship to AQP4 polarization in the perihematomal region. Immunofluorescence showed that GFAP immunoreactivity was markedly increased after ICH, accompanied by a significant increase in the proportion of C3^+^GFAP^+^ cells, indicating robust astrocyte activation in the perihematomal region with a shift toward a proinflammatory phenotype (Figure 4A–C). After HD‐tDCS treatment, the proportion of C3^+^GFAP^+^ cells was significantly reduced, whereas the proportion of S100A10^+^GFAP^+^ cells was markedly increased (Figure 4D,E), suggesting that HD‐tDCS treatment shifted astrocytes from a proinflammatory phenotype toward an anti‐inflammatory phenotype. Correlation analysis showed that the degree of AQP4 polarization was negatively associated with both mean GFAP fluorescence intensity and the proportion of C3^+^GFAP^+^ cells (Figure 4B,C).

**FIGURE 4:**
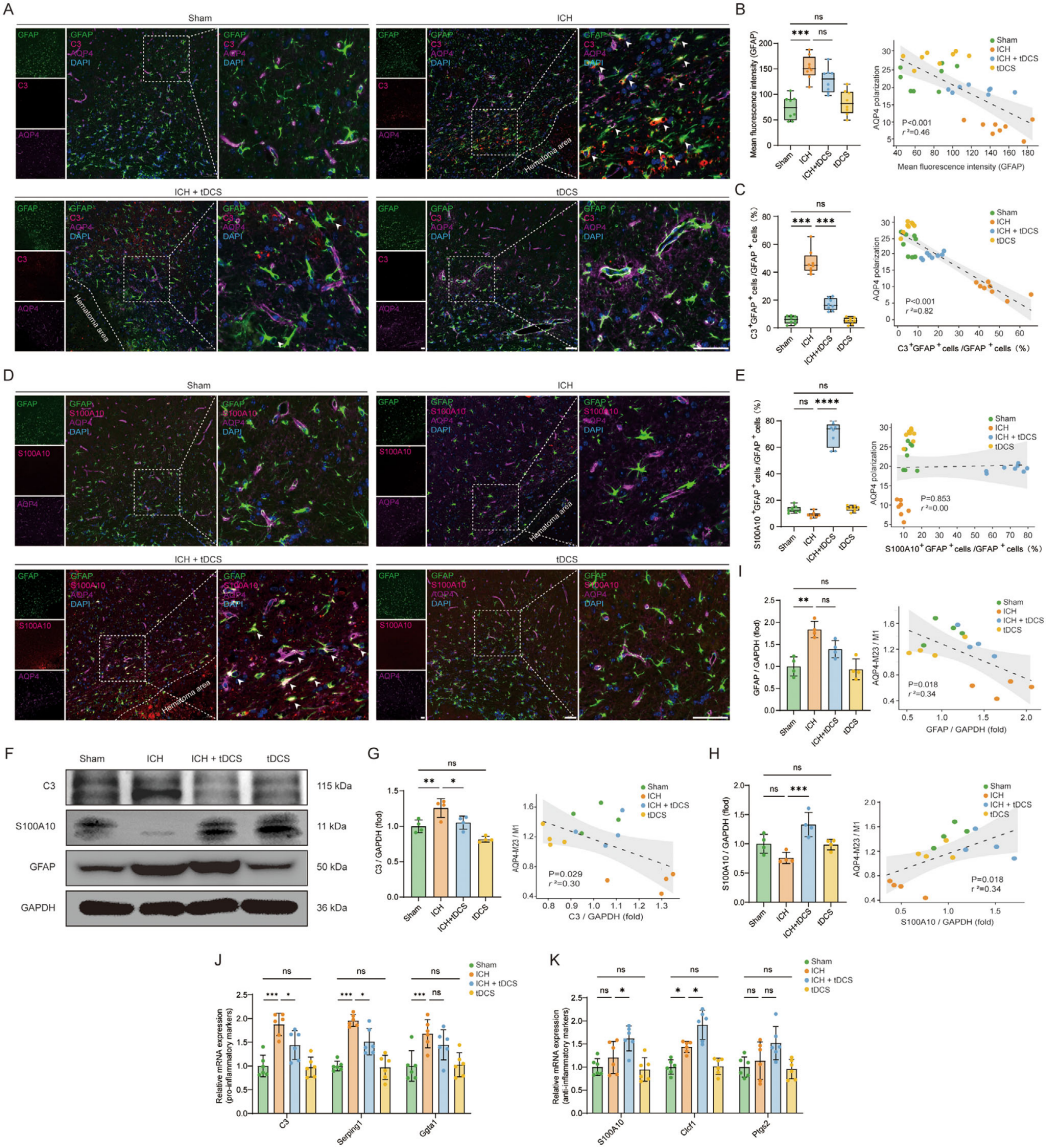
HD‐tDCS suppresses excessive astrocyte activation after ICH and promotes a phenotypic shift associated with restoration of AQP4 polarization. (A) Representative triple immunofluorescence images of GFAP (green), C3 (red), and AQP4 (purple) in the perihematomal region. Nuclei were counterstained with DAPI (blue) (scale bar, 50 µm). (B, C) Quantification of mean GFAP fluorescence intensity, the proportion of C3^+^GFAP^+^ cells among GFAP^+^ cells, and their correlations with AQP4 polarization (*n* = 8). (D) Representative triple immunofluorescence images of GFAP (green), S100A10 (red), and AQP4 (purple) in the perihematomal region. Nuclei were counterstained with DAPI (blue) (scale bar, 50 µm). (E) Quantification of the proportion of S100A10^+^GFAP^+^ cells among GFAP^+^ cells and its correlation with AQP4 polarization (*n* = 8). (F) Representative Western blot bands of C3, S100A10, and GFAP in perihematomal tissue. (G, H) Quantification of C3 and S100A10 protein expression and their correlations with the AQP4‐M23/AQP4‐M1 ratio (*n* = 8). (I, J) Quantification of mRNA expression of pro‐inflammatory markers (*C3, Serping1, and Ggta1*) and anti‐inflammatory markers (*S100a10, Clcf1, and Ptgs2*) (*n* = 6). Data are presented as mean ± SEM. ns, not significant; ^*^ *p* < 0.05, ^**^ *p* < 0.01, ^***^ *p* < 0.001, ^****^ *p* < 0.0001.

Western blot analysis further supported these findings. C3 and GFAP protein levels were both increased in ICH mice, whereas HD‐tDCS treatment significantly reduced C3 expression and increased S100A10 levels (Figure 4F–H). Further analysis showed that the AQP4‐M23/M1 ratio was negatively correlated with C3 and GFAP protein levels, but positively correlated with S100A10 expression (Figure 4G–I), suggesting that restoration of AQP4 polarization was associated with reduced reactive/ proinflammatory astrocytosis.

At the transcriptional level, the proinflammation‐associated markers C3 and Serping1 were significantly upregulated in perihematomal tissue from ICH mice, and their expression was partially reduced by HD‐tDCS treatment (Figure 4I). In parallel, HD‐tDCS treatment further increased the expression of the resist inflammation‐associated markers *S100a10* and *Clcf1* (Figure 4J). Taken together, these findings indicate that HD‐tDCS treatment suppresses excessive astrocyte reactivity and the C3‐associated proinflammatory phenotype in the perihematomal region after ICH, while promoting a phenotypic shift associated with restoration of AQP4 polarization. Supplementary immunofluorescence analysis further suggested that HD‐tDCS modulated perihematomal microglial activation, with corresponding changes in CD86‐ and CD206‐associated phenotypes (Figure S3). These findings indicate that the anti‐inflammatory effects of HD‐tDCS after ICH may involve coordinated regulation of multiple glial cell populations.

### 3.5 HD‐tDCS Up‐Regulates PPARγ Expression and Inhibition of NF‐κB/mTOR Activation in ICH Mice

To investigate the molecular basis underlying the protective effects of HD‐tDCS, we first performed pathway enrichment analysis of the differentially expressed genes and found that the PPAR signaling pathway was among the significantly enriched pathways (Figure 5A). GSEA further showed that this pathway was positively enriched in the ICH+tDCS group (NES = 1.65, adjusted *p* < 0.001), with *Pparg* identified among the core enriched genes (Figure 5B,C), suggesting a potential role for PPARγ in the effects of HD‐tDCS treatment.

**FIGURE 5:**
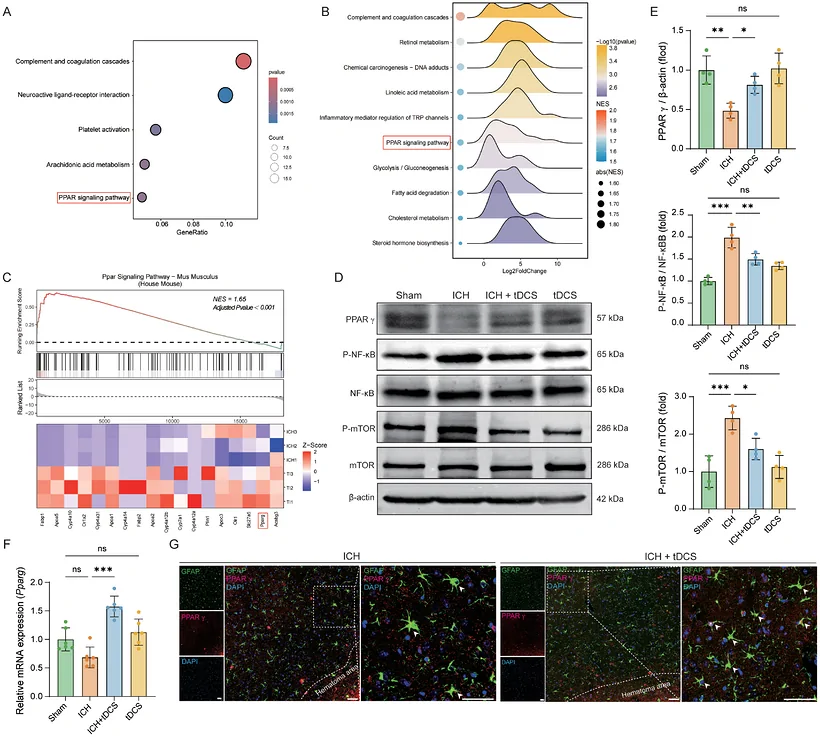
HD‐tDCS upregulates PPARγ expression and suppresses activation of the NF‐κB/mTOR pathway. (A) KEGG pathway enrichment analysis of differentially expressed genes, showing that the PPAR signaling pathway was one of the significantly enriched pathways. (B) Gene set enrichment analysis (GSEA) of the PPAR signaling pathway, showing positive enrichment after HD‐tDCS treatment. (C) Heatmap of the core enriched genes in the PPAR signaling pathway, indicating that *Pparg* was one of the core enriched genes. (D) Representative Western blot bands of PPARγ, p‐NF‐κB, NF‐κB, p‐mTOR, and mTOR protein expression in each group. (E) Quantitative analysis of PPARγ protein expression and the ratios of p‐NF‐κB to NF‐κB and p‐mTOR to mTOR in each group (*n* = 4). (F) Representative double immunofluorescence images of GFAP (green) and PPARγ (red) in the perihematomal region of the ICH and ICH+tDCS groups. Nuclei were counterstained with DAPI (blue) (scale bar, 50 µm). Merged images and higher‐magnification views show the distribution of PPARγ expression in GFAP‐positive astrocytes. Data are presented as mean ± SEM. ns, not significant; ^*^ *p* < 0.05, ^**^ *p* < 0.01, ^***^ *p* < 0.001, ^****^ *p* < 0.0001.

We next examined PPARγ and its related signaling pathways. Western blot analysis showed that, compared with the Sham group, PPARγ protein expression was markedly reduced in the ICH group, whereas the p‐NF‐κB/NF‐κB and p‐mTOR/mTOR ratios were significantly increased. Following HD‐tDCS treatment, PPARγ expression was restored, accompanied by reductions in both the p‐NF‐κB/NF‐κB and p‐mTOR/mTOR ratios (Figure 5D,E), suggesting that HD‐tDCS partially restores PPARγ expression while suppressing excessive activation of the NF‐κB/mTOR pathway.

Immunofluorescence further showed that PPARγ colocalized with GFAP‐positive astrocytes in the perihematomal region, and PPARγ immunoreactivity in astrocytes was increased in the ICH+tDCS group compared with the ICH group (Figure 5F). These findings suggest that the protective effects of HD‐tDCS after ICH may be mediated, at least in part, by upregulation of PPARγ in perihematomal astrocytes and concomitant suppression of NF‐κB/mTOR pathway activation.

### 3.6 Blocking or Knocking Down PPARγ Dampens HD‐tDCS Induced AQP4 Repolarization, Glymphatic Enhancement, and Neurological Recovery

To determine the role of PPARγ in mediating the protective effects of HD‐tDCS, we blocked PPARγ signaling using astrocyte‐specific knockdown with AAV9‐GFAP‐sh*Pparg* and pharmacologic inhibition with GW9662. The design of the astrocyte‐specific AAV9‐GFAP‐sh*Pparg* knockdown strategy and the in vivo distribution of viral expression in the basal ganglia are shown in Figure S4A. In mice treated with HD‐tDCS after ICH, AAV9‐GFAP‐sh*Pparg* significantly reduced PPARγ protein levels and decreased the AQP4‐M23/M1 ratio, without altering total AQP4 protein expression (Figure 6A,B). Concomitantly, C3 expression was increased, S100A10 expression was decreased, and the p‐NF‐κB/NF‐κB and p‐mTOR/mTOR ratios were elevated, indicating that PPARγ knockdown attenuated the regulatory effects of HD‐tDCS on astrocyte phenotype and inflammation‐related signaling (Figure 6A,B). Immunofluorescence further showed that PPARγ knockdown increased the proportion of C3^+^GFAP^+^ cells in the perihematomal region and markedly reduced AQP4 polarization (Figure 6C,D).

**FIGURE 6:**
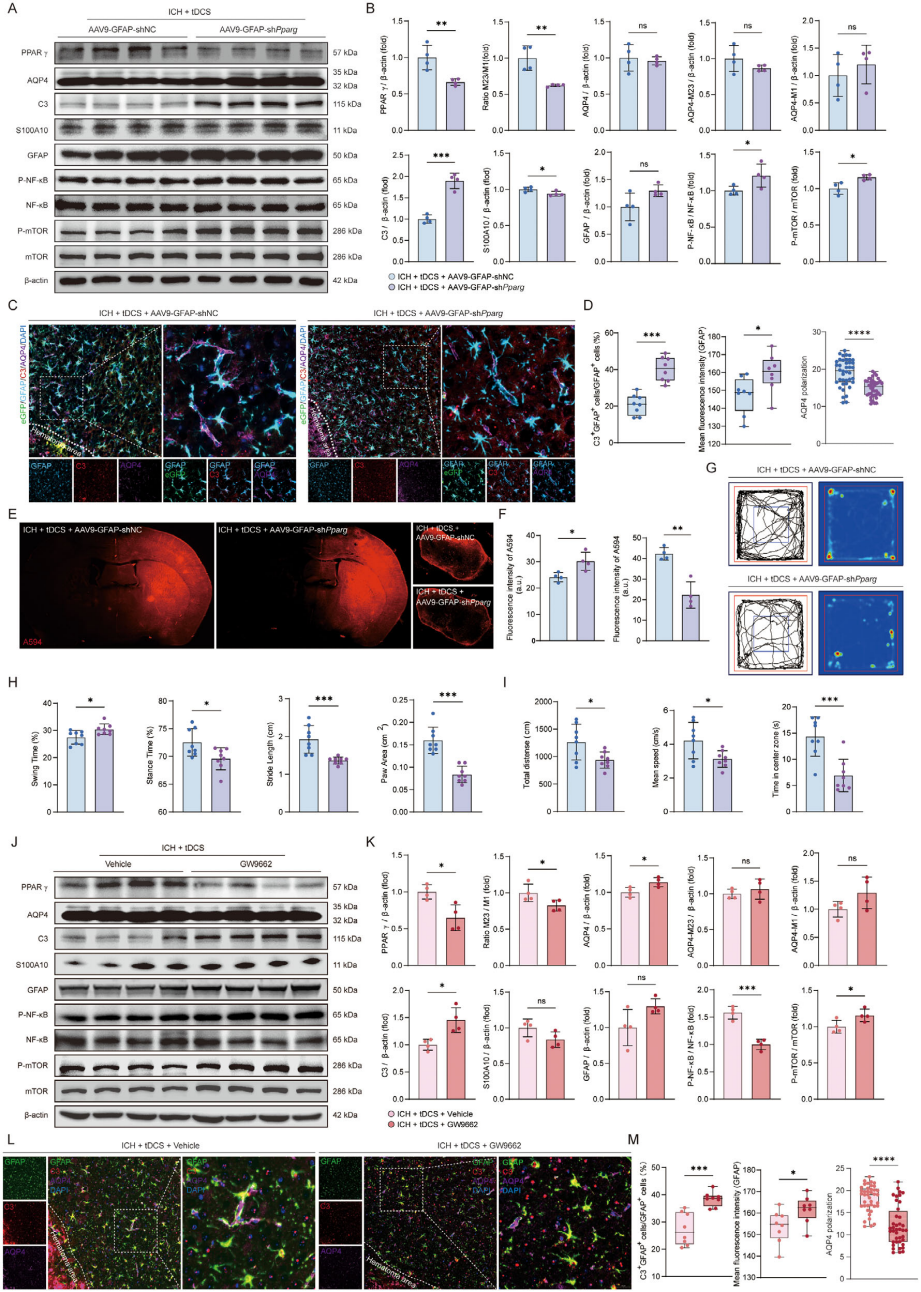
Blockade or knockdown of PPARγ attenuates HD‐tDCS‐induced AQP4 repolarization, glymphatic enhancement, and neurological recovery. (A) Representative Western blot bands from the ICH+tDCS+AAV9‐GFAP‐shNC and ICH+tDCS+AAV9‐GFAP‐shPparg groups. (B) Quantification of PPARγ, AQP4‐related proteins, C3, S100A10, GFAP, and NF‐κB/mTOR signaling in each group (*n* = 4). (C) Representative immunofluorescence images of GFAP (green), C3 (red), and AQP4 (purple) in the perihematomal region after PPARγ knockdown. Nuclei were counterstained with DAPI (blue) (scale bar, 50 µm). (D) Quantification of C3^+^GFAP^+^ cells, mean GFAP fluorescence intensity, and AQP4 polarization (*n* = 8). (E) Representative ex vivo A594 tracer images in the brain and deep cervical lymph nodes (dCLNs) from the ICH+tDCS+AAV9‐GFAP‐shNC and ICH+tDCS+AAV9‐GFAP‐sh*Pparg* groups. (F) Quantification of A594 fluorescence intensity in the brain and dCLNs (*n* = 4). (G) Representative open‐field trajectories and heat maps from each group. (H) Quantification of gait parameters, including swing time, stance time, stride length, and paw area (*n* = 8). (I) Quantification of total travel distance, mean speed, and time spent in the center zone in the open‐field test (*n* = 8). (J) Representative Western blot bands from the ICH+tDCS+Vehicle and ICH+tDCS+GW9662 groups. (K) Quantification of PPARγ, AQP4‐related proteins, C3, S100A10, GFAP, and NF‐κB/mTOR signaling in each group (*n* = 4). (L) Representative immunofluorescence images of GFAP (green), C3 (red), and AQP4 (purple) in the perihematomal region after GW9662 treatment. Nuclei were counterstained with DAPI (blue) (scale bar, 50 µm). (M) Quantification of C3^+^GFAP^+^ cells, mean GFAP fluorescence intensity, and AQP4 polarization (*n* = 8). Data are presented as mean ± SEM. ns, not significant; ^*^ *p* < 0.05, ^**^ *p* < 0.01, ^***^ *p* < 0.001, ^****^ *p* < 0.0001.

At the functional level, PPARγ knockdown markedly attenuated the HD‐tDCS‐induced improvement in glymphatic transport. At 2 h after injecting A594 into the striatum, PPARγ knockdown increased A594 retention in the brain and reduced drainage‐associated fluorescence, indicating impaired tracer efflux (Figure 6E,F). Behavioral testing showed that PPARγ knockdown reduced total distance traveled and mean speed in the open‐field test (Figure 6G,I). Gait analysis further showed prolonged swing time, shortened stance time, and reductions in stride length and paw area (Figure 6H), indicating that PPARγ knockdown markedly blunted the beneficial effects of HD‐tDCS on motor recovery.

Pharmacologic inhibition yielded similar results. Compared with the vehicle group, GW9662 treatment reduced PPARγ protein expression and the AQP4‐M23/M1 ratio, accompanied by increased C3 expression and a higher p‐NF‐κB/NF‐κB and p‐mTOR/mTOR ratio. In parallel, AQP4 polarization was reduced, whereas the proportion of C3^+^GFAP^+^ cells and GFAP immunoreactivity was increased (Figure 6J–M). Overall, whether achieved by genetic knockdown or pharmacologic inhibition, blockade of PPARγ markedly blunted HD‐tDCS‐induced AQP4 repolarization, glymphatic enhancement, and neurological recovery in ICH mice, indicating that PPARγ is a key mediator of the protective effects of HD‐tDCS.

### 3.7 Enhancing PPARγ Signaling Restores AQP4 Repolarization, Glymphatic Function, and Neurological Recovery in ICH Mice

We further used astrocyte‐specific PPARγ overexpression and GW1929 treatment to assess the effects of enhanced PPARγ signaling on AQP4 polarization, glymphatic function, and neurological outcomes in ICH mice, with the goal of further defining the mediating role of PPARγ in the protective effects of HD‐tDCS. The design of the astrocyte‐specific overexpression strategy and the in vivo distribution of viral expression in the basal ganglia are shown in Figure S4B. In ICH mice, AAV9‐GFAP‐*Pparg*‐OE markedly increased PPARγ protein expression and the AQP4‐M23/M1 ratio, without significantly affecting total AQP4 protein expression (Figure 7A,B). In parallel, C3 and GFAP expression decreased, S100A10 expression increased, and the p‐NF‐κB/NF‐κB and p‐mTOR/mTOR ratios were reduced (Figure 7A,B), suggesting that PPARγ overexpression suppresses the proinflammatory astrocytic response after ICH and promotes a phenotypic shift associated with restoration of AQP4 polarization.

**FIGURE 7:**
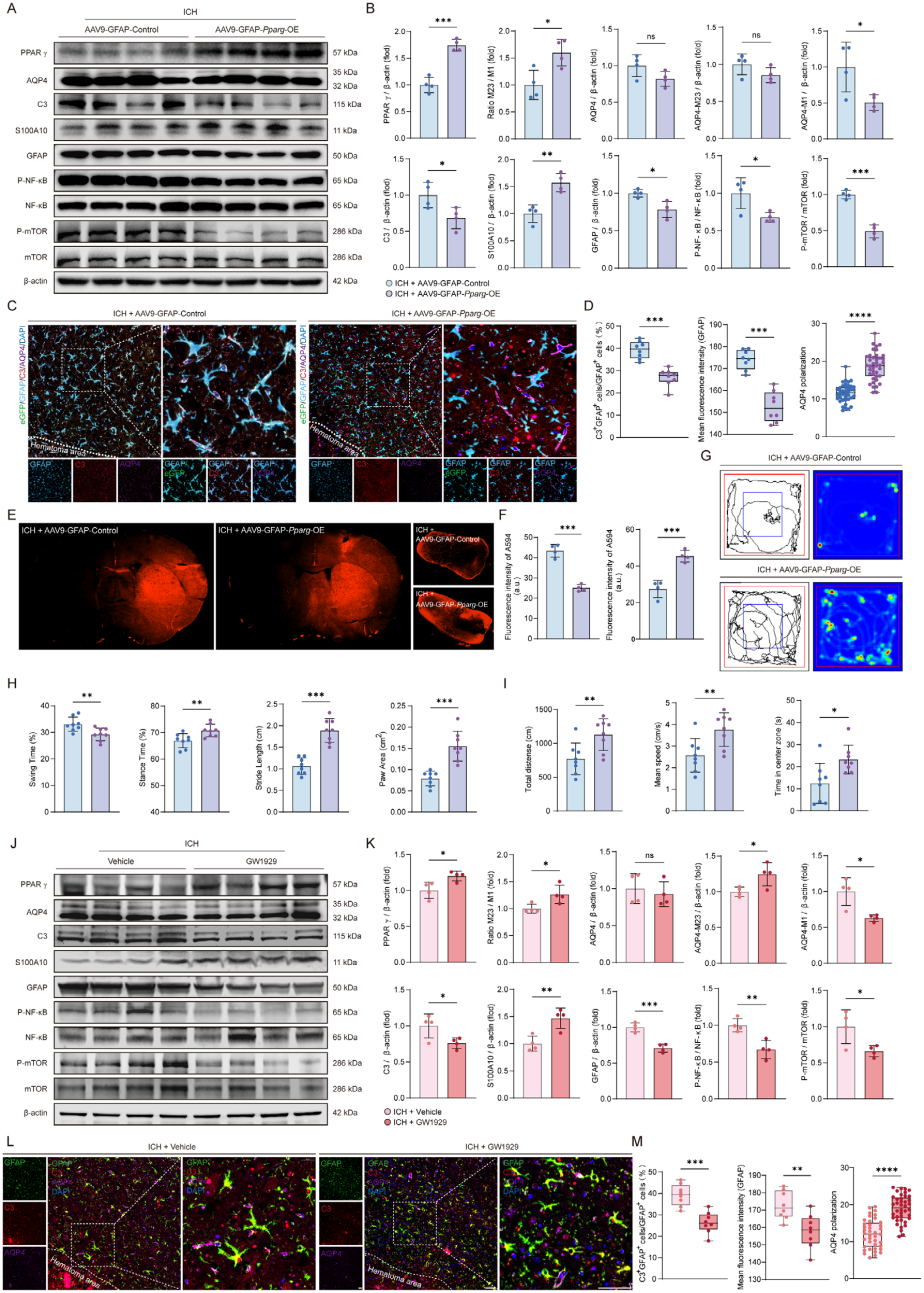
Enhancement of PPARγ signaling promotes AQP4 repolarization, glymphatic recovery, and neurological improvement. (A) Representative Western blot bands from the ICH+AAV9‐GFAP‐Control and ICH+AAV9‐GFAP‐*Pparg*‐OE groups. (B) Quantification of PPARγ, AQP4‐related proteins, C3, S100A10, GFAP, and NF‐κB/mTOR signaling in each group (*n* = 4). (C) Representative immunofluorescence images of GFAP (green), C3 (red), and AQP4 (purple) in the perihematomal region after PPARγ overexpression. Nuclei were counterstained with DAPI (blue) (scale bar, 50 µm). (D) Quantification of C3^+^GFAP^+^ cells, mean GFAP fluorescence intensity, and AQP4 polarization (*n* = 8). (E) Representative ex vivo A594 tracer images in the brain and deep cervical lymph nodes (dCLNs) from the ICH+AAV9‐GFAP‐Control and ICH+AAV9‐GFAP‐*Pparg*‐OE groups. (F) Quantification of A594 fluorescence intensity in the brain and dCLNs (*n* = 4). (G) Representative open‐field trajectories and heat maps from the ICH+AAV9‐GFAP‐Control and ICH+AAV9‐GFAP‐*Pparg*‐OE groups. (H) Quantification of gait parameters, including swing time, stance time, stride length, and paw area (*n* = 8). (I) Quantification of total travel distance, mean speed, and time spent in the center zone in the open‐field test (*n* = 8). (J) Representative Western blot bands from the ICH+Vehicle and ICH+GW1929 groups. (K) Quantification of PPARγ, AQP4‐related proteins, C3, S100A10, GFAP, and NF‐κB/mTOR signaling in each group (*n* = 4). (L) Representative immunofluorescence images of GFAP (green), C3 (red), and AQP4 (purple) in the perihematomal region after GW1929 treatment. Nuclei were counterstained with DAPI (blue) (scale bar, 50 µm). (M) Quantification of C3^+^GFAP^+^ cells, mean GFAP fluorescence intensity, and AQP4 polarization (*n* = 8). Data are presented as mean ± SEM. ns, not significant; ^*^ *p* < 0.05, ^**^ *p* < 0.01, ^***^ *p* < 0.001, ^****^ *p* < 0.0001.

Immunofluorescence further showed that PPARγ overexpression reduced both the proportion of C3^+^GFAP^+^ cells and GFAP signal intensity in the perihematomal region, while significantly increasing AQP4 polarization (Figure 7C,D). Functionally, PPARγ overexpression decreased intracerebral A594 retention and increased drainage‐associated fluorescence, indicating improved glymphatic clearance (Figure 7E,F). In parallel, open‐field testing showed increases in total distance traveled and mean speed (Figure 7G,I). Gait analysis further showed shortened swing time, prolonged stance time, and increases in stride length and paw area (Figure 7H), indicating that PPARγ overexpression improved spontaneous activity and motor deficits in ICH mice.

As a pharmacological approach to activate PPARγ signaling, we further treated ICH mice with GW1929, a selective PPARγ agonist. Compared with the vehicle group, GW1929 treatment increased PPARγ protein expression and the AQP4‐M23/M1 ratio, accompanied by reduced C3 expression, increased S100A10 expression, and lower p‐NF‐κB/NF‐κB and p‐mTOR/mTOR ratios (Figure 7J,K). Immunofluorescence further showed that GW1929 reduced the proportion of C3^+^GFAP^+^ cells and GFAP signal intensity in the perihematomal region while increasing AQP4 polarization (Figure 7L,M). These findings indicate that enhancement of PPARγ signaling reshapes astrocyte status in ICH mice, promotes AQP4 repolarization, and improves glymphatic function and neurological outcomes. The similarity of these effects to the protective phenotype induced by HD‐tDCS further supports a mediating role for PPARγ in the actions of HD‐tDCS.

### 3.8 HD‐tDCS Improves CBF After ICH, Whereas PPARγ Inhibition Does not Alter CBF Changes

Given that hemodynamic factors may contribute to glymphatic transport, we next examined whether HD‐tDCS altered CBF after ICH using laser speckle contrast imaging. CBF was assessed in the ipsilateral ROI on Day 1 and Day 3 in the Sham, ICH, ICH+tDCS, and tDCS groups. Compared with the Sham group, the tDCS group showed increased CBF in the ipsilateral ROI on both Day 1 and Day 3. In the ICH model, CBF in the ipsilateral ROI was significantly reduced on Day 3, whereas HD‐tDCS treatment increased CBF compared with the ICH group at this time point (Figure S6A,B). These findings suggest that HD‐tDCS may improve the hemodynamic microenvironment after ICH.

We further asked whether the PPARγ‐dependent effect of HD‐tDCS on glymphatic function was associated with changes in CBF. To address this question, CBF was measured in the ICH+tDCS+vehicle and ICH+tDCS+GW9662 groups before treatment and on Day 1 and Day 3 after ICH. No significant difference in CBF was observed between the two groups at any examined time point (Figure S6C,D). Together with the finding that PPARγ inhibition impaired HD‐tDCS‐induced glymphatic efflux (Figure 6E,F and Figure S6E,F), these results suggest that improved CBF may represent an important hemodynamic component of HD‐tDCS‐mediated regulation after ICH, whereas PPARγ‐dependent restoration of glymphatic function is unlikely to be explained solely by changes in CBF.

### 3.9 HD‐tDCS Modulates MMP‐9/β‐DG‐Associated Anchoring Remodeling Through PPARγ‐Dependent Astrocyte‐State

Reactive astrocytes after ICH can release inflammation‐related molecules such as MMP‐9. These molecules may cleave β‐dystroglycan (β‐DG), disrupt normal AQP4 anchoring, and contribute to endothelial junction disruption, leukocyte infiltration, and increased blood‐brain barrier permeability, thereby aggravating perihematomal edema. Because perivascular AQP4 localization depends on anchoring and extracellular matrix‐associated structures, we further examined whether HD‐tDCS‐induced, PPARγ‐dependent astrocyte‐state remodeling was associated with MMP‐9/β‐DG‐related perivascular remodeling. Compared with Sham mice, ICH mice showed increased active MMP‐9 and enhanced β‐DG cleavage, indicating disruption of the perivascular anchoring environment after hemorrhagic injury. HD‐tDCS reduced MMP‐9 activation and β‐DG cleavage while preserving intact β‐DG (Figure S7A,B). Moreover, under ICH+tDCS conditions, PPARγ inhibition partially reversed the HD‐tDCS‐induced reductions in active MMP‐9 and cleaved β‐DG, whereas pharmacological activation of PPARγ produced a similar protective pattern (Figure S7C–E). These findings suggest that suppression of MMP‐9‐associated β‐DG degradation may be one mechanism through which PPARγ‐dependent astrocyte‐state remodeling creates a perivascular microenvironment permissive for AQP4 repolarization.

## 4 Discussion

The current study demonstrates ICH is commonly accompanied by glymphatic dysfunction, which may delay the clearance of hematoma and perihematomal edema [8, 9, 11]. In this study, we systematically evaluated the effects of HD‐tDCS on hematoma and edema resolution after ICH and explored the underlying mechanisms. Our findings showed that HD‐tDCS suppressed the proinflammatory activation of perihematomal astrocytes, reshaped AQP4 isoform composition, restored perivascular AQP4 polarization, and improved glymphatic drainage. Further analysis suggested that PPARγ‐associated astrocyte remodeling may be an important regulatory component of this process. These findings suggest that modulation of glymphatic dysfunction may represent a promising component of strategies to facilitate hematoma and edema clearance after ICH, and that HD‐tDCS may hold translational promise as a therapeutic approach.

The glymphatic system is a perivascular fluid exchange network distributed throughout the brain that provides a pathway for cerebrospinal fluid influx and metabolic waste clearance in a variety of neurological injuries. This process is driven primarily by arterial pulsation, depends on AQP4 localized to astrocytic endfeet, and is further modulated by sleep and circadian rhythms [22]. Notably, ICH is associated with impaired perivascular AQP4 polarization and disrupted glymphatic drainage, whereas interventions targeting AQP4 polarization have been shown to promote the clearance of hematoma and perihematomal edema [9, 23]. Previous studies have also shown that certain neuromodulatory approaches, such as transcranial ultrasound, can facilitate hematoma clearance after intracerebral hemorrhage by improving AQP4 polarization and glymphatic circulation [24]. More recently, tDCS has been reported to enhance cerebrospinal fluid–interstitial fluid exchange in healthy mice without significantly affecting AQP4 polarization. However, because AQP4 is more severely depolarized under pathological conditions such as ICH [14], whether tDCS can restore AQP4 polarization and improve glymphatic function in this setting remains unclear, and its therapeutic potential has yet to be established.

In the present study, we assessed the effect of HD‐tDCS on glymphatic function in ICH mice by examining both interstitial solute clearance and CSF influx. Consistent with earlier findings [9], ICH mice exhibited marked glymphatic dysfunction during the acute phase, characterized by reduced cerebrospinal fluid influx, impaired interstitial fluid drainage to the deep cervical lymph nodes, and delayed tracer clearance within perihematomal perivascular spaces. HD‐tDCS partially restored these abnormalities. Dynamic contrast‐enhanced MRI further confirmed that HD‐tDCS enhanced cerebrospinal fluid–interstitial fluid exchange in ICH mice, with more pronounced recovery in the cortex, hippocampus, and thalamus, suggesting a degree of regional specificity in its effects on cerebrospinal fluid circulation. When considered together with the MRI findings showing accelerated resolution of hematoma and perihematomal edema on days 5 and 7 after HD‐tDCS treatment, these results suggest that restoration of glymphatic function may represent an important mechanism underlying enhanced fluid clearance after ICH. Given that efficient removal of hematoma and edema is critical for limiting secondary brain injury and improving outcome, and that HD‐tDCS also improved motor performance in ICH mice, we speculate that restoration of glymphatic function may contribute to, rather than solely mediate, the neuroprotective effects of HD‐tDCS after ICH.

Our findings identify astrocyte phenotype‐associated AQP4 polarization as a critical target through which HD‐tDCS preserves glymphatic function in ICH mice. AQP4, a member of the aquaporin family, is expressed in astrocytes, the Purkinje cell layer of the cerebellum, the hypothalamus, and other brain regions [25]. Glymphatic function is highly dependent on AQP4 polarization, defined as the preferential enrichment of AQP4 at perivascular astrocytic endfeet [6, 7]. Our results showed that AQP4 polarization was markedly impaired in ICH mice. After HD‐tDCS treatment, perivascular AQP4 localization was improved. These findings suggest that the therapeutic effects of HD‐tDCS are mediated, at least in part, by restoring AQP4 polarization and function. This finding is consistent with previous studies in ischemic stroke [21], which showed that perivascular AQP4 polarization is disrupted after stroke and that loss or mislocalization of AQP4 exacerbates edema formation or delays its resolution. However, most previous studies were limited to AQP4 knockout models or pharmacologic suppression of AQP4 expression as a means of modulating AQP4 localization and glymphatic function [9, 26]. Notably, when AQP4 expression was inhibited, the resulting changes in AQP4 localization and glymphatic function were not consistent across studies. This inconsistency may reflect differential effects on distinct AQP4 isoforms. In the present study, we found that the ratio between AQP4‐M23 and AQP4‐M1 was reduced in ICH mice, which may contribute to abnormal AQP4 localization after hemorrhagic stroke. Orthogonal arrays of particles are closely associated with AQP4 polarization, and the AQP4‐M23 isoform is critical for maintaining their structural integrity [27]. Following HD‐tDCS treatment, the ratio between AQP4‐M23 and AQP4‐M1 increased, which expanded perivascular AQP4 coverage and improved glymphatic function. These findings suggest that the relative balance between AQP4 isoforms may be more important for glymphatic function than total AQP4 expression. A cohort study suggested that aberrant astrocyte activation may represent an important intermediary in glymphatic dysfunction [28]. Our study further showed that HD‐tDCS suppressed astrocyte activation and the shift toward a proinflammatory phenotype in the perihematomal region after ICH in mice. At the same time, the degree of AQP4 polarization was negatively correlated with markers of astrocyte activation. These findings suggest that the restorative effect of HD‐tDCS on glymphatic function after ICH may not result from a direct action on AQP4 itself. Rather, HD‐tDCS may improve astrocyte reactivity in the perihematomal region and thereby create a cellular microenvironment more favorable for perivascular AQP4 repolarization. Further studies are needed to determine whether HD‐tDCS can improve glymphatic dysfunction after human intracerebral hemorrhage by suppressing proinflammatory astrocyte activation.

We further identified PPARγ, a ligand‐activated nuclear receptor transcription factor, as a key mediator of the suppression of aberrant astrocyte activation and AQP4 mislocalization. In the present study, as well as in several previous reports [29, 30, 31], PPARγ expression was reduced in reactive astrocytes with increased AQP4 expression after brain injury, and this reduction was associated with astrocyte dysregulation and neuroinflammation. Our RNA sequencing data showed that PPARγ expression was significantly upregulated in the brains of ICH mice after HD‐tDCS treatment. Functional studies further showed that astrocyte‐specific knockdown of PPARγ markedly attenuated the ability of HD‐tDCS to restore perivascular AQP4 localization, glymphatic function, and motor performance. Previous studies have shown that PPARγ suppresses astrocyte overactivation and neuroinflammation, at least in part, through inhibition of NF‐κB and mTOR signaling [31, 32]. Consistent with this, knockdown or pharmacologic inhibition of PPARγ in HD‐tDCS‐treated ICH mice led to further activation of the NF‐κB and mTOR pathways and was accompanied by a shift of astrocytes toward a proinflammatory phenotype. In contrast, PPARγ overexpression in ICH mice suppressed NF‐κB and mTOR signaling, restored AQP4 polarization and glymphatic function, and improved motor outcomes. Our additional CBF analysis suggests that HD‐tDCS may improve the hemodynamic microenvironment after ICH, which could contribute to glymphatic restoration. However, PPARγ inhibition did not affect HD‐tDCS‐associated CBF changes, despite impairing glymphatic efflux. Together, these findings support the involvement of PPARγ‐associated astrocyte remodeling in HD‐tDCS‐related suppression of pathological astrocyte activation, improvement of AQP4 localization, and recovery of glymphatic function, while not excluding contributions from broader neurovascular and inflammatory mechanisms. Further studies are needed to define the mechanisms linking PPARγ‐dependent astrocyte remodeling to perivascular AQP4 repolarization and to determine the translational relevance of this pathway in clinical samples and long‐term follow‐up after ICH.

Beyond AQP4 localization, astrocytes contribute to the progression of hematoma and perihematomal edema after ICH through multiple mechanisms. For example, astrocytic endfeet may form a valve‐like structure that helps regulate unidirectional fluid movement [33], and dynamic changes in astrocyte volume may influence glymphatic transport by altering perivascular and interstitial spaces [34]. Astrocytes may also influence glymphatic function indirectly by modulating local cerebral blood flow through neurovascular coupling [35, 36]. Our findings provide complementary evidence that astrocyte‐state remodeling may further affect glymphatic function by modulating AQP4 polarization and the perivascular anchoring microenvironment. Future studies using ChIP‐qPCR, CUT&Tag, single‐cell transcriptomics, spatial transcriptomics, and broader analyses of anchoring‐complex components such as α‐syntrophin, dystroglycan, agrin, laminin, and related extracellular matrix molecules will be needed to clarify how distinct astrocyte states regulate AQP4 localization after ICH.

In addition, statistical limitations should also be noted. Sensitivity analyses indicated that several key animal‐level endpoints were adequately powered to detect large biologically meaningful effects, whereas RNA‐seq with *n* = 3/group and selected MRI endpoints with *n* = 3/group were interpreted as exploratory or supportive. Accordingly, our central conclusions are based on convergent evidence from glymphatic imaging, AQP4 localization analysis, protein validation, glial phenotyping, PPARγ gain‐ and loss‐of‐function experiments, and behavioral assessments rather than on any single small‐sample dataset.

From a translational perspective, HD‐tDCS represents a noninvasive and repeatable neuromodulatory approach with potential clinical applicability in ICH. However, translation to human ICH will require careful consideration of stimulation timing, lesion location, individual electric field distribution, hemodynamic effects, and safety in the setting of acute brain injury. Future studies should incorporate longer‐term behavioral and neuroplasticity assessments in animal models, together with clinical neuroimaging and functional outcome evaluation, to determine whether the glymphatic‐ and astrocyte‐related effects observed in mice can be reproduced in patients with ICH. Another issue that should be considered is how HD‐tDCS delivered at the cortical surface may influence pathological changes located in the basal ganglia. Although current‐distribution modeling was not performed in the present study, previous human and animal studies suggest that the electric field generated by transcranial electrical stimulation is expected to be concentrated mainly in superficial cortical regions [12, 13]. Future studies integrating current‐distribution modeling, cell‐type‐specific analyses, and spatial transcriptomics will be needed to define the brain regions, cellular targets, and signaling pathways through which HD‐tDCS regulates astrocyte remodeling and glymphatic recovery after ICH.

In conclusion, our study demonstrates that HD‐tDCS promotes the resolution of hematoma and perihematomal edema after intracerebral hemorrhage. This effect may be related to restoration of astrocyte phenotype–associated AQP4 polarization and improvement of glymphatic function. We further showed that HD‐tDCS upregulated PPARγ expression in ICH mice and suppressed aberrant astrocyte activation and AQP4 depolarization, whereas PPARγ overexpression produced similar protective effects. These findings suggest that enhancement of glymphatic function may represent an effective strategy for promoting hematoma and edema clearance after intracerebral hemorrhage, and that HD‐tDCS holds potential as a therapeutic approach.

## Author Contributions

ZML, YMZ, and QQT designed the experiments, performed the whole study, conducted data analysis, drafted and revised the manuscript. ZML, QY, and YMZ participated in animal model construction, grouping, and behavioral tests. ZML, QQT, and ZQ participated in the molecular experiment and immunofluorescence experiment. SYZ, ZTG, and HZ participated in the design and modification of the experiment. QL provided funding and conception of this study. All authors agreed on the submitted version of the manuscript. ZML, YMZ, and QQT contributed equally to this work. All authors read and approved the final manuscript.

## Funding

This study was supported by the National Natural Science Foundation of China (No. 82471368), the National College Students’ Innovation and Entrepreneurship Training Program (No. 202510366038), the Graduate Research and Practice Innovation Program of Anhui Medical University (No. YJS20240105), the Excellent Research and Innovation Team Project of Anhui Province (2024AH010014) and the Clinical and Translational Research Project of Anhui Province (202427b10020090).

## Ethics

All procedures complied with institutional and national regulations and were approved by the Experimental Animal Ethics Committee of Anhui Medical University (Approval No. LLSC20242252).

## Conflicts of Interest

The authors declare no conflicts of interest.

## Supporting information




**Supporting File**: advs76660‐sup‐0001‐SuppMat.docx.

## Data Availability

The data that support the findings of this study are available from the corresponding author upon reasonable request.
