# Supplementary material for: HD‐tDCS Restores Perivascular AQP4 Polarization via PPARγ Modulation to Enhance Glymphatic Clearance After Intracerebral Hemorrhage in Mice
Source: Adv Sci (Weinh). 2026 Jul 17:e76660. Online ahead of print. doi: 10.1002/advs.76660 (PMC13379215; doi:10.1002/advs.76660)
Supplement: Supplementary file 1 — Supporting File: advs76660‐sup‐0001‐SuppMat.docx. [file ADVS-9999-e76660-s001.docx]

**
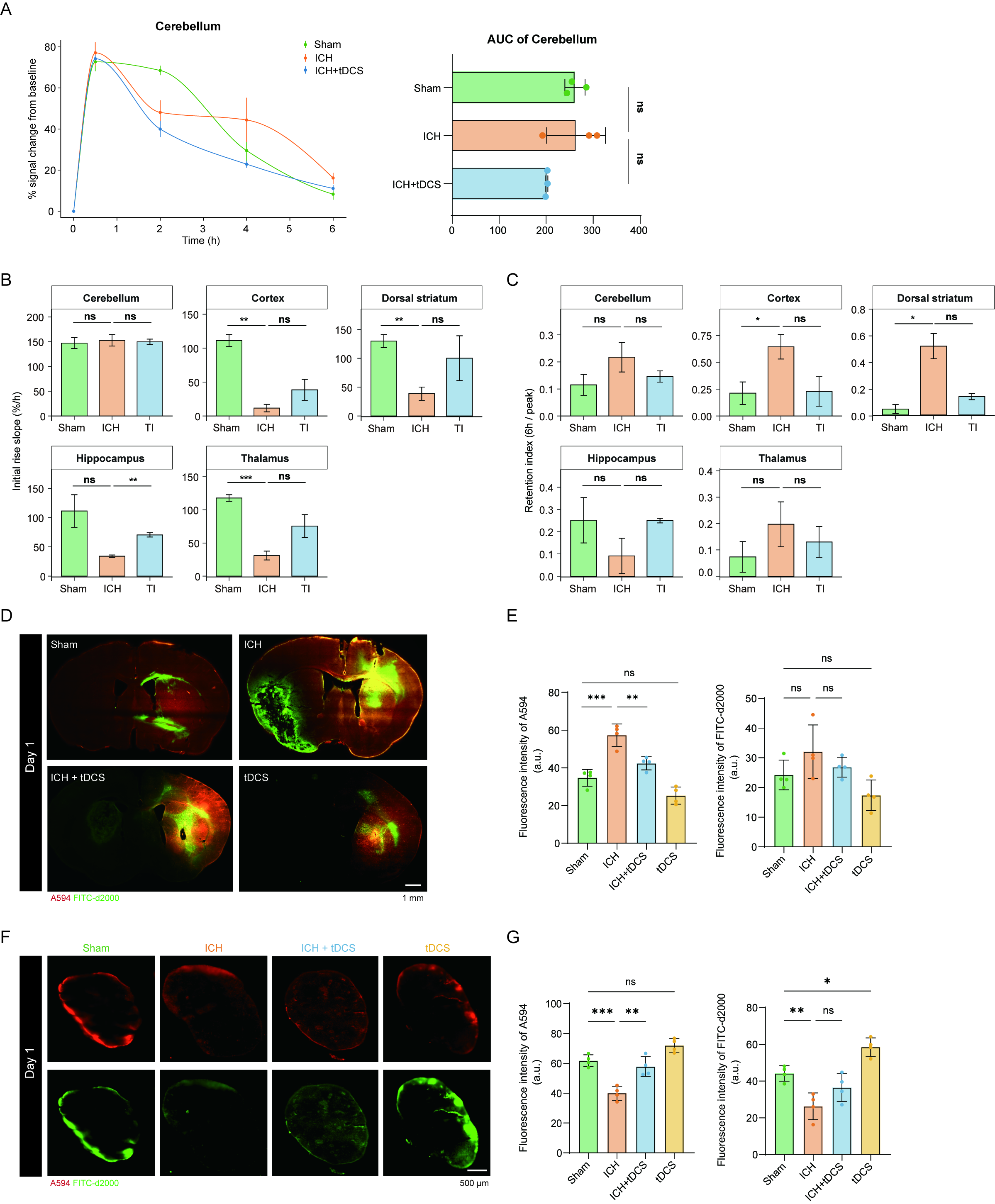
**

**Supplementary Figure 1 | Regional glymphatic transport kinetics and Day 1 tracer efflux after ICH and HD-tDCS treatment.** (A) Time–signal curve and AUC quantification of Gd-DTPA enhancement in the cerebellum among the Sham, ICH, and ICH+tDCS groups (n = 3). (B) Quantification of the initial rise slope in the cerebellum, cortex, dorsal striatum, hippocampus, and thalamus among the Sham, ICH, and ICH+tDCS groups (n = 3). (C) Quantification of the retention index in the cerebellum, cortex, dorsal striatum, hippocampus, and thalamus among the Sham, ICH, and ICH+tDCS groups (n = 3). (D) Representative fluorescence images of brain sections showing A594 and FITC-d2000 signals in the Sham, ICH, ICH+tDCS, and tDCS groups on Day 1. (E) Quantification of A594 and FITC-d2000 fluorescence intensity in brain sections among the Sham, ICH, ICH+tDCS, and tDCS groups on Day 1 (n = 4). (F) Representative fluorescence images of dCLNs showing A594 and FITC-d2000 signals in the Sham, ICH, ICH+tDCS, and tDCS groups on Day 1. (G) Quantification of A594 and FITC-d2000 fluorescence intensity in dCLNs among the Sham, ICH, ICH+tDCS, and tDCS groups on Day 1 (n = 4). ns, not significant; *P < 0.05; **P < 0.01; ***P < 0.001.

**
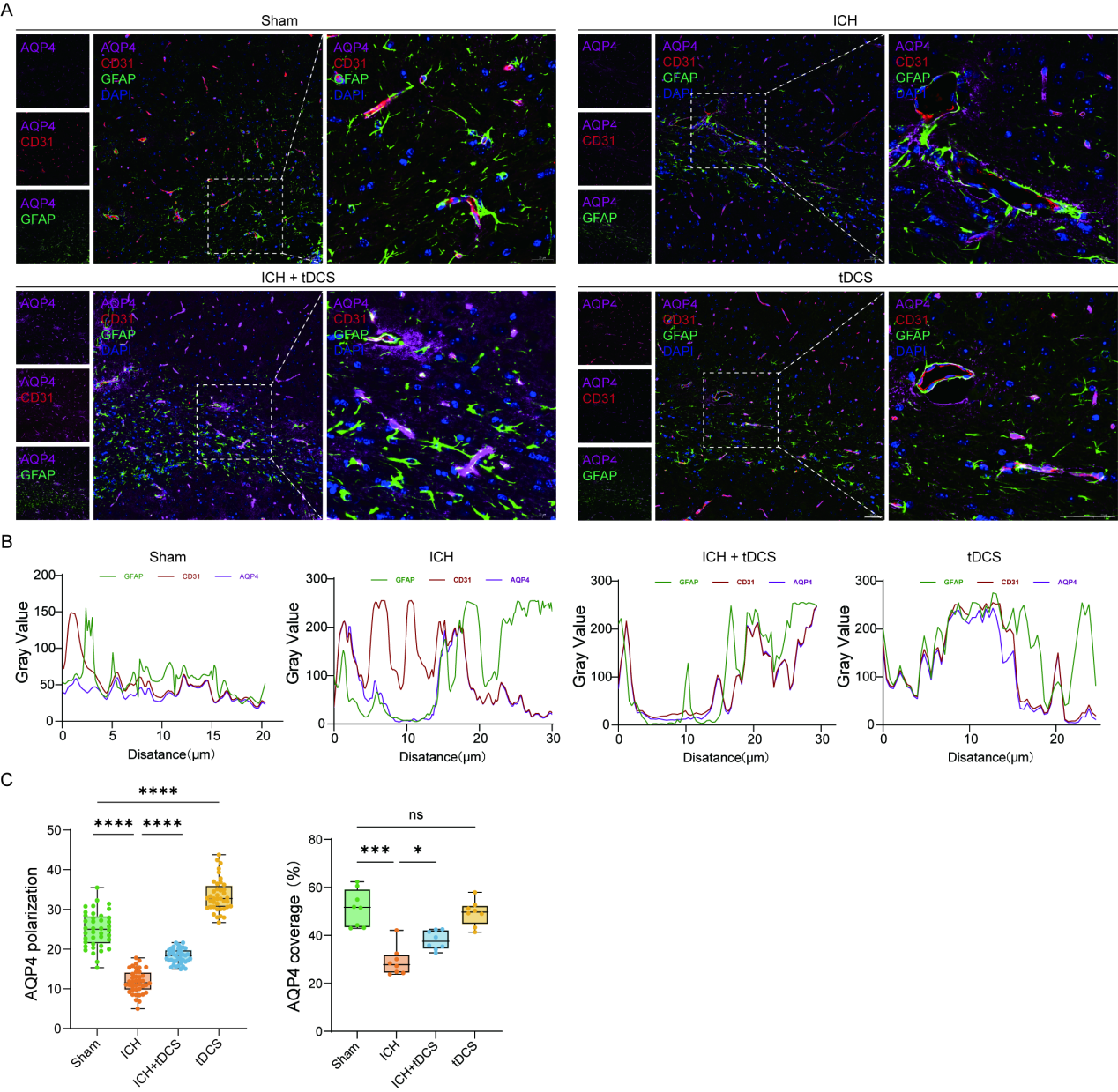
**

**Supplementary Figure 2 | HD-tDCS restores perivascular AQP4 localization after ICH.** (A) Representative immunofluorescence images showing AQP4, CD31, GFAP, and DAPI staining in the Sham, ICH, ICH+tDCS, and tDCS groups. Enlarged images show the perivascular distribution of AQP4 around CD31-positive vessels and GFAP-positive astrocytic endfeet. (B) Representative line-scan profiles showing the spatial relationship among AQP4, CD31, and GFAP fluorescence signals in the indicated groups. (C) Quantification of AQP4 polarization and AQP4 perivascular coverage among the Sham, ICH, ICH+tDCS, and tDCS groups (n = 8). Data are presented as box plots. Statistical analysis was performed using one-way ANOVA followed by multiple-comparison tests. ns, not significant; *P < 0.05; ***P < 0.001; ****P < 0.0001.

**
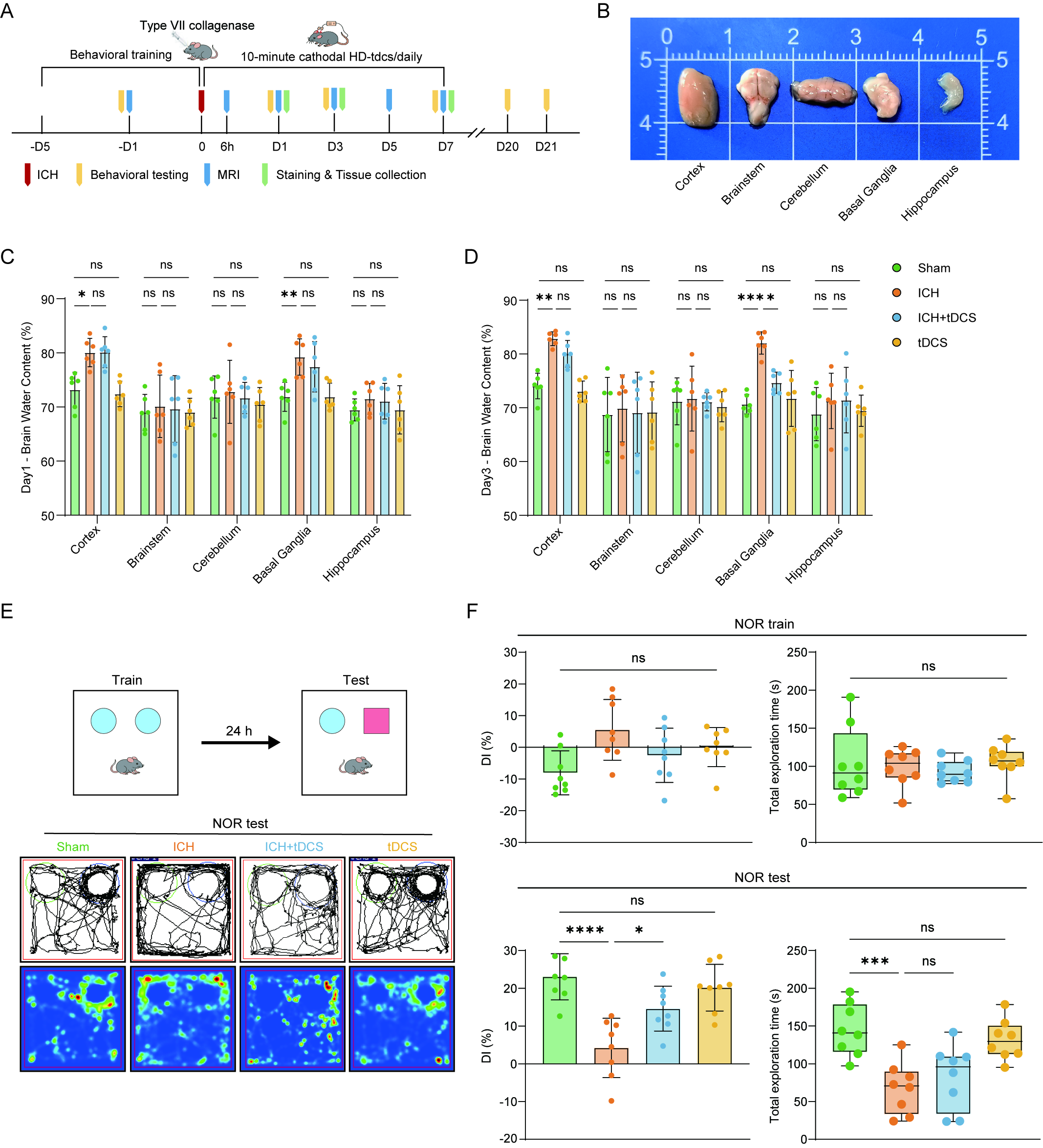
**

**Supplementary Figure 3 | Assessment of brain water content and recognition memory after ICH and HD-tDCS treatment.** (A) Schematic illustration of the experimental timeline. (B) Representative image showing the dissected brain regions used for brain water content measurement, including the cortex, brainstem, cerebellum, basal ganglia, and hippocampus. (C-D) Quantification of brain water content in different brain regions on Day 1and Day3 after ICH (n = 6). (E) Schematic diagram of the novel object recognition (NOR) test and representative movement trajectories and heatmaps during the NOR test in the Sham, ICH, ICH+tDCS, and tDCS groups. (F) Quantification of the discrimination index (DI) and total exploration time during the NOR training and test phases among the Sham, ICH, ICH+tDCS, and tDCS groups (n = 8). Statistical analysis was performed using one-way ANOVA followed by multiple-comparison tests. ns, not significant; *P < 0.05; **P < 0.01; ***P < 0.001; ****P < 0.0001.

**
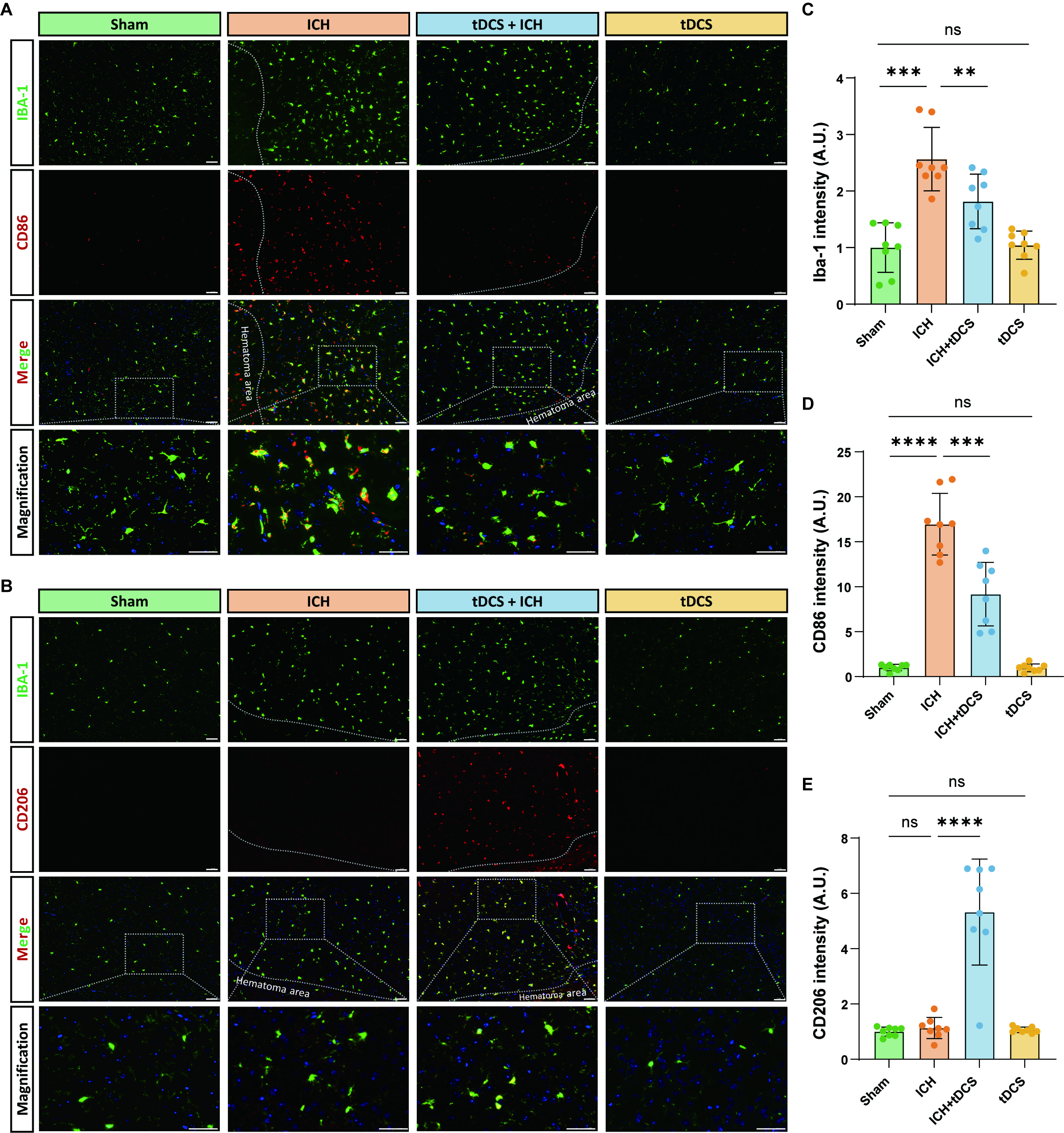
**

**Supplementary Figure 4 | HD-tDCS modulates microglial activation after ICH.** (A) Representative immunofluorescence images showing IBA-1 and CD86 staining. Enlarged images show microglial activation and CD86-associated signals in the perihematomal region. (B) Representative immunofluorescence images showing IBA-1 and CD206 staining. Enlarged images show microglial activation and CD206-associated signals in the perihematomal region. (C) Quantification of IBA-1 fluorescence intensity among the Sham, ICH, ICH+tDCS, and tDCS groups (n = 8). (D) Quantification of CD86 fluorescence intensity among the indicated groups (n = 8). (E) Quantification of CD206 fluorescence intensity among the indicated groups (n = 8). Statistical analysis was performed using one-way ANOVA followed by multiple-comparison tests. ns, not significant; **P < 0.01; ***P < 0.001; ****P < 0.0001.

**
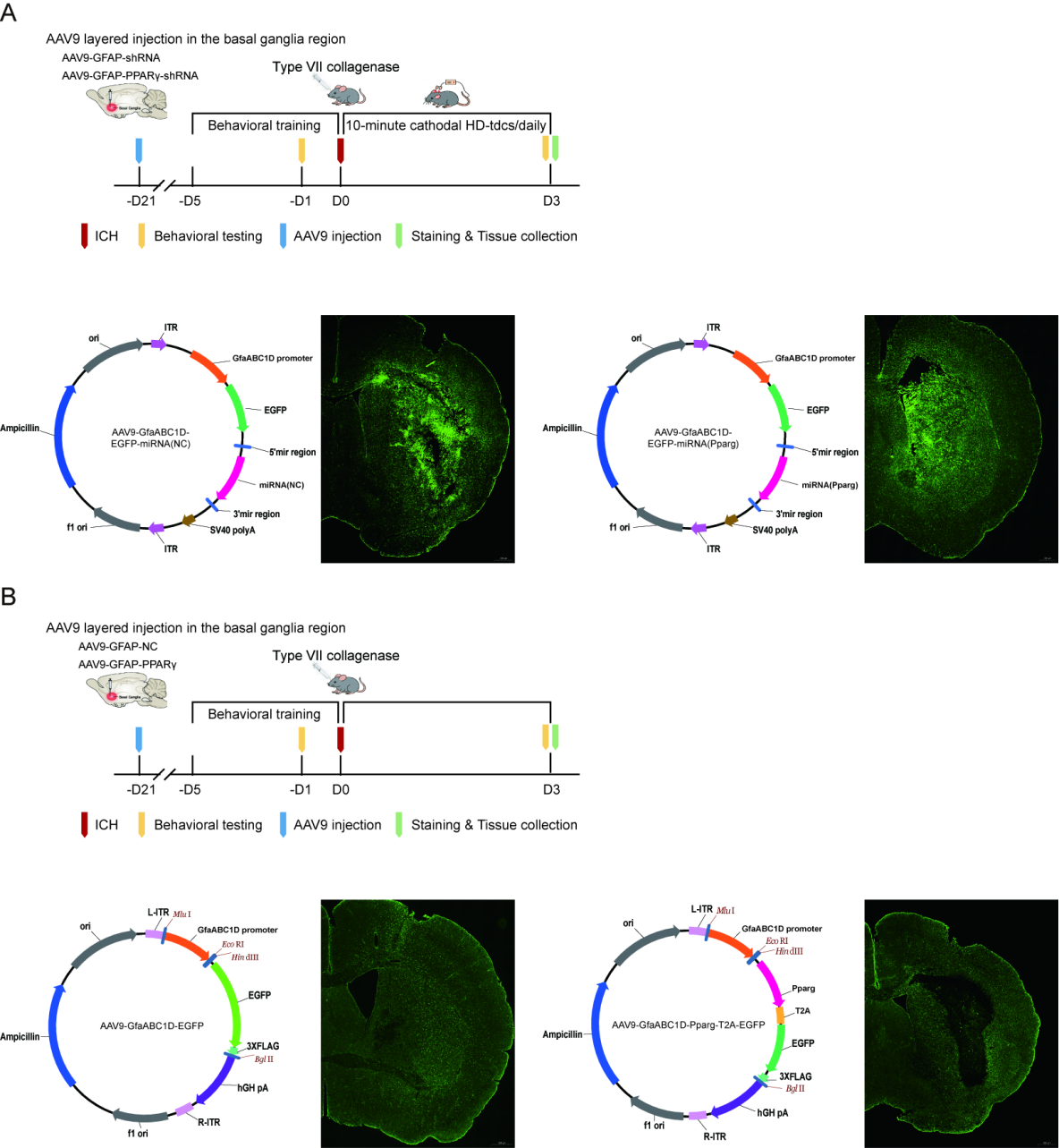
**

**Supplementary Figure 5 | AAV9-mediated astrocyte-targeted PPARγ knockdown and overexpression strategies in the basal ganglia.** (A) Experimental timeline, vector design, and representative fluorescence images showing the in vivo distribution of AAV9-GFAP-shNC and AAV9-GFAP-sh*Pparg* in the basal ganglia region. AAV9 vectors were injected before ICH induction, followed by daily HD-tDCS intervention and tissue collection on Day 3. (B) Experimental timeline, vector design, and representative fluorescence images showing the in vivo distribution of AAV9-GFAP-NC and AAV9-GFAP-*Pparg* in the basal ganglia region. Green fluorescence indicates viral expression in the injected brain region.

**
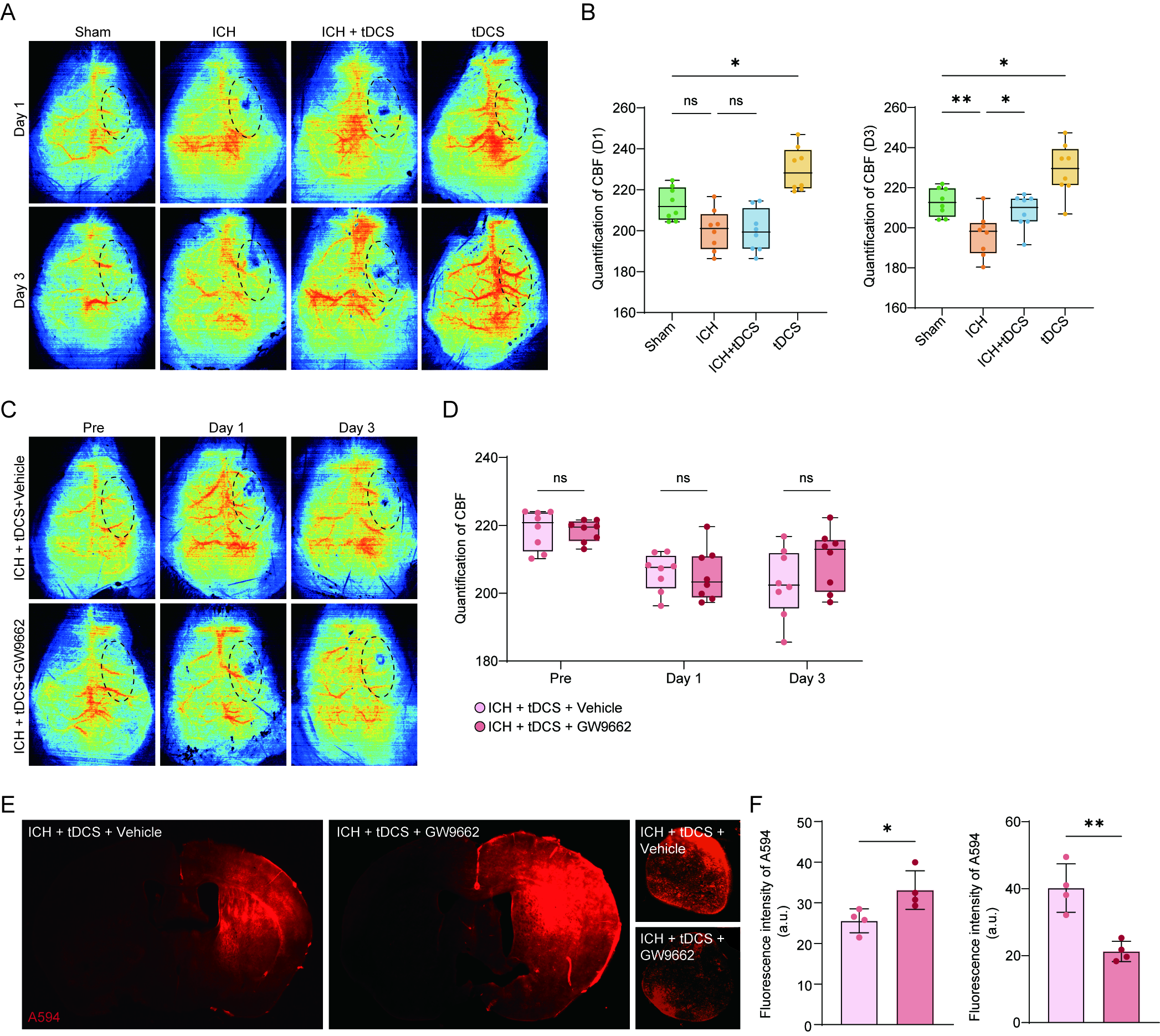
**

**Supplementary Figure 6 | HD-tDCS improves CBF after ICH, whereas PPARγ inhibition does not alter CBF changes but impairs glymphatic efflux.** (A) Representative laser speckle contrast imaging of CBF in the Sham, ICH, ICH+tDCS, and tDCS groups on Day 1 and Day 3. (B) Quantification of CBF in the ipsilateral ROI among the Sham, ICH, ICH+tDCS, and tDCS groups on Day 1 and Day 3 (n = 8; one-way ANOVA followed by multiple-comparison tests). (C) Representative laser speckle contrast imaging of CBF in the ICH+tDCS+vehicle and ICH+tDCS+GW9662 groups before treatment, on Day 1, and on Day 3. (D) Quantification of CBF in the ipsilateral ROI between the ICH+tDCS+vehicle and ICH+tDCS+GW9662 groups before treatment, on Day 1, and on Day 3 (n = 8; two-way repeated-measures ANOVA followed by multiple-comparison tests). (E) Representative fluorescence images of brain sections and dCLNs showing A594 signals in the ICH+tDCS+vehicle and ICH+tDCS+GW9662 groups. (F) Quantification of A594 fluorescence intensity in brain sections and dCLNs between the ICH+tDCS+vehicle and ICH+tDCS+GW9662 groups (n = 4; t-test). ns, not significant; *P < 0.05; **P < 0.01.

**
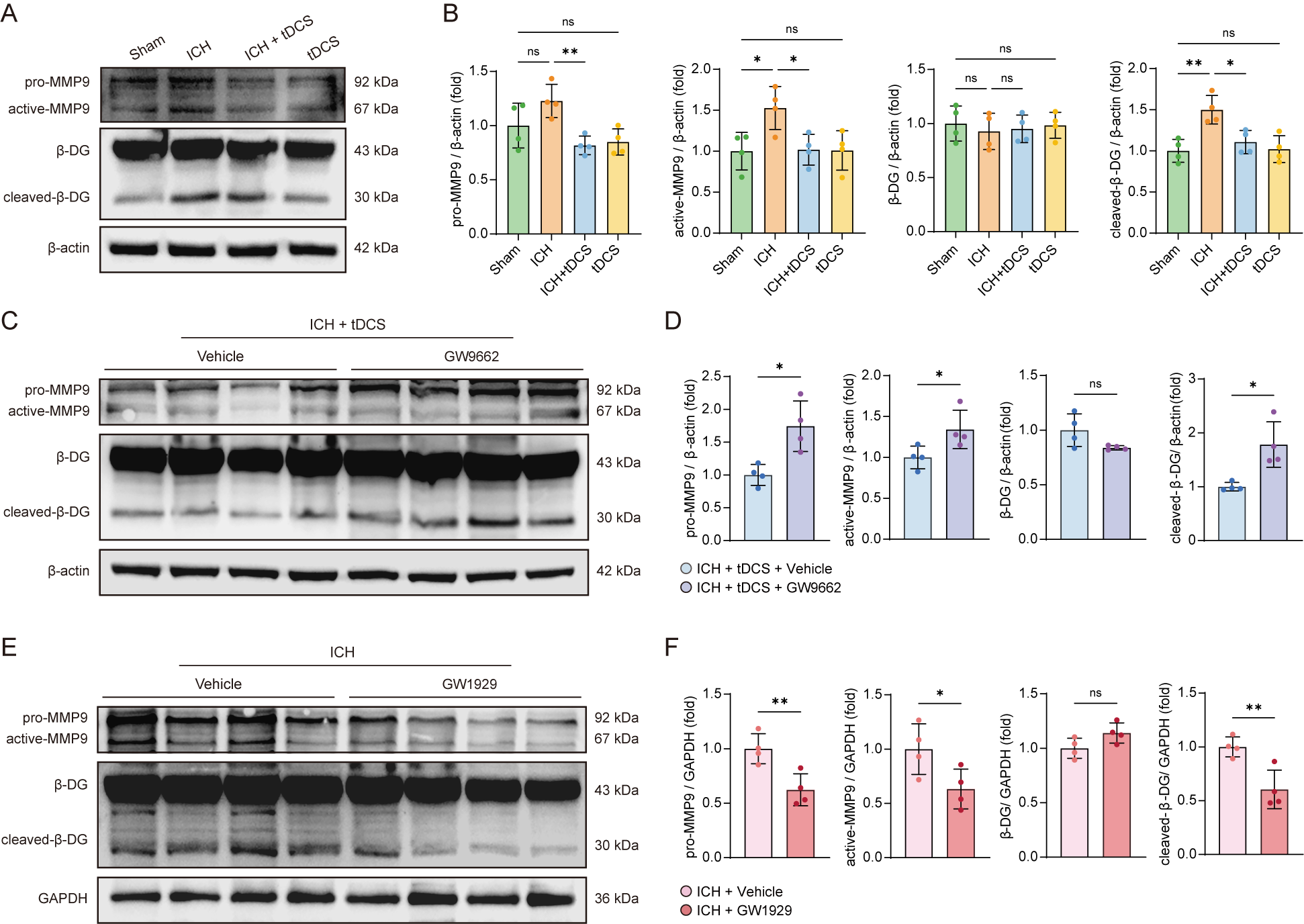
**

**Supplementary Figure 7 | PPARγ signaling regulates the MMP-9/β-DG anchoring-related pathway after ICH.** (A) Representative western blots of pro-MMP-9, active MMP-9, intact β-DG, and cleaved β-DG in the Sham, ICH, ICH+tDCS, and tDCS groups. (B) Quantification of pro-MMP-9, active MMP-9, intact β-DG, and cleaved β-DG protein levels among the Sham, ICH, ICH+tDCS, and tDCS groups (n = 4; one-way ANOVA followed by multiple-comparison tests). (C) Representative western blots of pro-MMP-9, active MMP-9, intact β-DG, and cleaved β-DG in the ICH+tDCS+vehicle and ICH+tDCS+GW9662 groups. (D) Quantification of pro-MMP-9, active MMP-9, intact β-DG, and cleaved β-DG protein levels between the ICH+tDCS+vehicle and ICH+tDCS+GW9662 groups (n = 4; t-test). (E) Representative western blots of pro-MMP-9, active MMP-9, intact β-DG, and cleaved β-DG in the ICH+vehicle and ICH+GW1929 groups. (F) Quantification of pro-MMP-9, active MMP-9, intact β-DG, and cleaved β-DG protein levels between the ICH+vehicle and ICH+GW1929 groups (n = 4; t-test). β-actin or GAPDH was used as the loading control. ns, not significant; *P < 0.05; **P < 0.01.

**Supplementary Table 1. Sequences of primers for qPCR analysis.**

| Gene | Forward (5′→3′) | Reverse (5′→3′) |
| --- | --- | --- |
| *Pparg* | AACCCACAACCAAATCCACAC | ATCACGGAGAGGTCCACAGA |
| *Aqp4* | ATCAGCATCGCTAAGTCCGTC | GAGGTGTGACCAGGTAGAGGA |
| *Aqp4-m1* | CTCCCAGTGTACTGGAGCCCG | TGGTGACTCCCAATCCTCCAAC |
| *Aqp4-m23* | GGAAGGCTAGGTTGGTGACTTC | TGGTGACTCCCAATCCTCCAAC |
| *C3* | GGCTTGTAGGGTGACACTCAG | GGGGTCACGATCAGTGTTT |
| *S100a10* | CCTCTGGCTGGGACAAAAT | CTGCTCACAAGAAGCAGTGG |
| *Serping1* | ACAGCCCCCTCTGAATTCTT | GGATGCTCTCCAAGTTTGCTC |
| *Clcf1* | CTTCAATCCTCCTCGACTGG | TACGTCGGAGTTCAGCTGTG |
| *Ggta1* | GTGAACAGCATGAGGGGTTT | GTTTTGTTGCCTCTGGGTGT |
| *Ptgs2* | GCTGTACAAGCAGTGGCAAA | CCCCAAAGATAGCATCTGGA |

| **Supplementary Table S2. Sample-size justification and sensitivity analysis for representative endpoints** | | | | |
| --- | --- | --- | --- | --- |
| **Category / endpoint** | **Biological n/group** | **Observed \|d\|** | **Minimum detectable d at 80% power** | **Interpretation** |
| RNA-seq | 3 | N/A | 3.07 | Exploratory discovery screen |
| CE-MRI AUC, Cortex | 3 | 3.58 | 3.07 | Large observed effect, but exploratory/supportive due to n=3 |
| CE-MRI AUC, Thalamus | 3 | 2.78 | 3.07 | Below 80% detectable range; supportive, not standalone |
| Two-photon tracer clearance, 120 min | 5 | 1.74 | 2.02 | Below 80% detectable range; supportive, not standalone |
| Ex vivo brain tracer retention | 4 | 3.71 | 2.38 | Large functional support |
| dCLN tracer drainage | 4 | 6.11 | 2.38 | Large functional support |
| Edema volume, D7 | 4 | 2.39 | 2.38 | Approximately at threshold; supportive |
| Edema volume, D5 | 4 | 1.87 | 2.38 | Below 80% detectable range; supportive, not standalone |
| Hematoma volume, D7 | 4 | 2.0646 | 2.38 | Below 80% detectable range; supportive, not standalone |
| Hematoma volume, D5 | 4 | 1.9997 | 2.38 | Below 80% detectable range; supportive, not standalone |
| rADC, D3 | 4 | 1.78 | 2.38 | Below 80% detectable range; supportive, not standalone |
| MLS, D7 | 4 | 1.239 | 2.38 | Below 80% detectable range; supportive, not standalone |
| MLS, D5 | 4 | 2.59 | 2.38 | Large functional support |
| Rotarod, D7 | 10 | 1.9655 | 1.32 | Large functional support |
| Rotarod, D5 | 10 | 4.85 | 1.32 | Large functional support |
| mNSS, D7 | 10 | 1.57 | 1.32 | Large functional support |
| mNSS, D5 | 10 | 1.57 | 1.32 | Large functional support |
| NOR discrimination index | 8 | 1.49 | 1.51 | Approximately at threshold; supportive |
| Total distance, D7 | 8 | 1.72 | 1.51 | Large functional support |
| Total distance, D5 | 8 | 2.84 | 1.51 | Large functional support |
| PPARγ knockdown:Total distance | 8 | 1.30 | 1.51 | Approximately at threshold; supportive |
| PPARγ overexpression:Total distance | 8 | 1.53 | 1.51 | Large functional support |
| Paw area, D7 | 8 | 3.25 | 1.51 | Large functional support |
| Paw area, D3 | 8 | 2.45 | 1.51 | Large functional support |
| PPARγ knockdown:Paw area | 8 | 2.76 | 1.51 | Large functional support |
| PPARγ overexpression:Paw area | 8 | 3.12 | 1.51 | Large functional support |
| Swing Time (%), D7 | 8 | 2.42 | 1.51 | Large functional support |
| Swing Time (%), D5 | 8 | 2.00 | 1.51 | Large functional support |
| PPARγ knockdown:Swing Time (%) | 8 | 1.31 | 1.51 | Approximately at threshold; supportive |
| PPARγ overexpression:Swing Time (%) | 8 | 1.51 | 1.51 | Approximately at threshold; supportive |
| Stance Time (%), D7 | 8 | 2.42 | 1.51 | Large functional support |
| Stance Time (%), D5 | 8 | 2.00 | 1.51 | Large functional support |
| PPARγ knockdown:Stance Time (%) | 8 | 1.31 | 1.51 | Approximately at threshold; supportive |
| PPARγ overexpression:Stance Time (%) | 8 | 1.51 | 1.51 | Approximately at threshold; supportive |
| PPARγ knockdown:Stride Length (cm) | 8 | 3.48 | 1.51 | Large mechanistic support |
| PPARγ overexpression:Stride Length (cm) | 8 | 2.118 | 1.51 | Large mechanistic support |
| AQP4 coverage, ipsilateral hemisphere | 8 | 1.62 | 1.51 | Large mechanistic support |
| AQP4-M23/M1 ratio | 4 | 4.22 | 2.38 | Large mechanistic support |
| PPARγ protein expression | 4 | 3.24 | 2.38 | Large mechanistic support |
| PPARγ knockdown: AQP4-M23/M1 | 4 | 3.15 | 2.38 | Large mechanistic support |
| PPARγ knockdown: dCLN drainage | 4 | 3.98 | 2.38 | Large functional support |
| Sensitivity analyses were performed using a conservative two-sided independent-samples t-test framework with α = 0.05, power = 0.80, and 1:1 group allocation. The minimum detectable Cohen’s d represents the smallest standardized effect size that could be detected with 80% power under the actual biological sample size. Observed \|d\| values were calculated from representative pairwise contrasts. Biological n refers to the number of animals per group; multiple fields, sections, or ROIs from the same animal were treated as technical sampling and averaged to generate one animal-level value before statistical testing. | | | | |
